# Changes in the Oligodendrocyte Progenitor Cell Proteome with Ageing

**DOI:** 10.1074/mcp.RA120.002102

**Published:** 2020-11-23

**Authors:** Alerie G. de la Fuente, Rayner M.L. Queiroz, Tanay Ghosh, Christopher E. McMurran, Juan F. Cubillos, Dwight E. Bergles, Denise C. Fitzgerald, Clare A. Jones, Kathryn S. Lilley, Colin P. Glover, Robin J.M. Franklin

**Affiliations:** 1 Wellcome-MRC Cambridge Stem Cell Institute, Jeffrey Cheah Biomedical Centre, University of Cambridge, United Kingdom; 2 Cambridge Centre for Proteomics, Department of Biochemistry, University of Cambridge, United Kingdom; 3 Respiratory, Inflammation and Autoimmunity, MedImmune Ltd., Granta Park, United Kingdom; 4 Department of Medicine, University of Cambridge School of Clinical Medicine, Addenbrooke's Hospital, Hills Road, Cambridge, United Kingdom; 5 The Solomon H. Snyder Department of Neuroscience, Johns Hopkins University School of Medicine, Baltimore, USA; 6 John Hopkins University, Kavli Neuroscience Discovery Institute, USA; 7 Wellcome-Wolfson Institute for Experimental Medicine, Queen's University Belfast, United Kingdom; 8 Oncology Early Clinical Projects, Oncology R &D, AstraZeneca, Melbourn Science Park, Melbourn, Hertfordshire, United Kingdom

**Keywords:** Aging, stem cells, neurodegenerative diseases, neurobiology, progenitor cells, multiple sclerosis, myelin, oligodendrocyte, regeneration

## Abstract

Following central nervous system (CNS) demyelination, adult oligodendrocyte progenitor cells (OPCs) can differentiate into new myelin-forming oligodendrocytes in a regenerative process called remyelination. Although remyelination is very efficient in young adults, its efficiency declines progressively with ageing. Here we performed proteomic analysis of OPCs freshly isolated from the brains of neonate, young and aged female rats. Approximately 50% of the proteins are expressed at different levels in OPCs from neonates compared with their adult counterparts. The amount of myelin-associated proteins, and proteins associated with oxidative phosphorylation, inflammatory responses and actin cytoskeletal organization increased with age, whereas cholesterol-biosynthesis, transcription factors and cell cycle proteins decreased. Our experiments provide the first ageing OPC proteome, revealing the distinct features of OPCs at different ages. These studies provide new insights into why remyelination efficiency declines with ageing and potential roles for aged OPCs in other neurodegenerative diseases.

## Teaser

Oligodendrocyte progenitor cell (OPC)-mediated remyelination declines with ageing. We have determined the proteome of neonatal, young and aged rat OPCs using quantitative MS-proteomics. We identify increases in myelin proteins as well as changes in inflammation, cholesterol biosynthesis and protein homeostasis pathways in OPCs with ageing. These data provide a resource to study age-associated changes in OPCs that may contribute to the age-related decrease in oligodendrogliogenesis.

## Graphical Abstract

**Figure:**
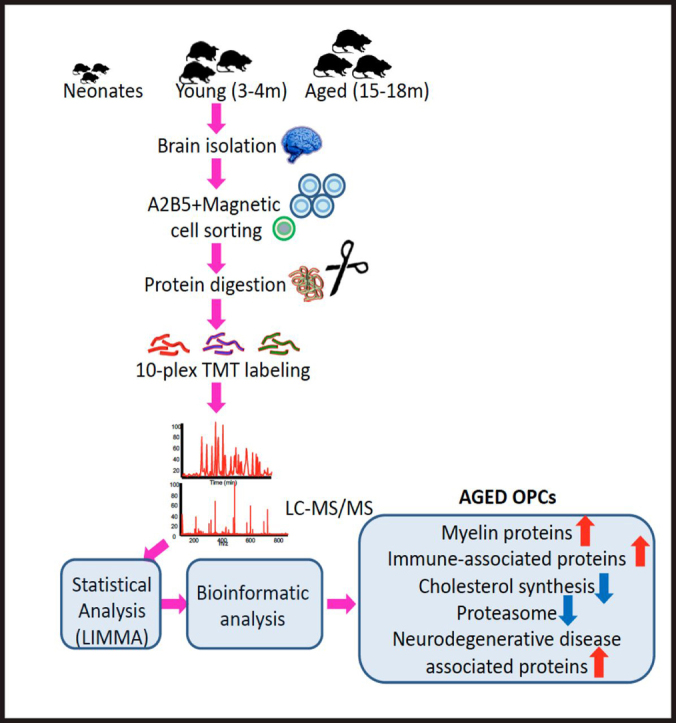

**Highlights**

• Quantitative proteome of neonatal, young, and aged OPCs.
• 50% of the proteome is differentially expressed between neonatal and adult OPCs.
• Myelin proteins are increased, and cholesterol synthesis proteins decreased with age.
• Proteins associated with other neurodegenerative diseases are increased in aged OPCs.

Oligodendrocyte progenitor cells (OPCs) are the cells responsible for generating new myelin sheath-forming oligodendrocytes in the regenerative process of remyelination. Remyelination restores rapid conduction of action potentials and metabolic support to axons, and prevents axonal degeneration in chronic demyelinating diseases such as multiple sclerosis (MS) (1). During remyelination, adult OPCs activate, divide, migrate and differentiate into myelin forming oligodendrocytes in response to a demyelinating insult (2, 3, 4). Remyelination is a highly efficient process in young adults: however, as with all regenerative processes, its efficiency declines progressively with ageing (5). A combination of environmental changes, such as the capacity of phagocytic cells to remove myelin debris (6, 7, 8), as well as cell-intrinsic epigenetic changes (9), contribute to the age-associated decline in remyelination efficiency (10). This decline is thought to be a major determinant of disease progression in MS patients, and is proposed to be a significant factor in the transition into the largely untreatable progressive phase of the disease (11).

Several brain related RNA-Seq datasets have been published recently (2, 12, 13), including recent RNA datasets comparing OPCs at different ages (14, 15). However, the correlation between RNA expression and the expression of proteins can be low (16, 17): hence, there is clear need for a comprehensive quantitative analysis of ageing OPCs proteome. Other publications have addressed the proteome of OPCs derived from human embryonic stem cells (18), the proteome of OPCs during differentiation to oligodendrocytes (19) and the proteome of various CNS specific cell types in the mouse brain, including oligodendrocytes (17). However, none of these have compared the changes in OPCs associated with ageing, which is a key factor in remyelination failure during disease progression (5, 10). To address the changes in protein abundance observed within ageing OPCs, we performed a comprehensive quantitative proteomic analysis of neonatal, young adult and aged rat OPCs. The OPC proteomes we have delineated represent a new and important resource to understand the age-related changes that underlie remyelination failure, which will help develop identify remyelination-enhancing approaches and define the role of OPCs in other age-related diseases.

## MATERIALS AND METHODS

##### Oligodendrocyte Progenitor Cell Isolation

OPC were isolated from neonatal (P0-P2), 3–4 months old and 15–18 months old Sprague-Dawley rats. Briefly, rats were euthanized by overdose of intraperitoneal injection of Pentoject and the decapitated following schedule 1 procedures (Animal Scientific Procedure Act 1986). The brains were removed from the skull and put in to “Hibernate A” (prepared in-house) (20). Meninges were removed and the brains were cut into small pieces (about 1 mm^3^). The brains were digested with a papain solution containing papain (33U/ml) and DNase (0.04 mg/ml) in Hibernate A for 40 min at 35 °C. Following tissue digestion papain was washed out with Hank's Balanced Salt Solution (HBSS) (−/−) (Gibco, Altrincham, UK) via centrifugation. The tissue was then triturated with a polished glass Pasteur pipette in Hibernate A with 1×B27 (Gibco) and 2 mm sodium pyruvate (Gibco). After trituration, the single cell suspension was passed through a 70 μm strainer and centrifuged for 20 min at 800 × *g* in a 22.5% Percoll (GE Healthcare, Amersham, UK) solution with DMEM F12(Gibco). Following centrifugation Percoll was removed by aspiration and the cells were washed with HBSS (−/−) (Gibco). Cells were incubated with anti-A2B5 antibody (Millipore, Dorset, UK, MAB312) for 25 min at 4 °C (2 μg of antibody per 10^7^ cells) in wash buffer (Miltenyi Wash Buffer [MWB] = 0.5% BSA, 2 mm sodium pyruvate and 2 mm EDTA in 1× PBS). Excess antibody was washed with HBSS (−/−) and cells were incubated with secondary IgM-microbeads (Miltenyi Biotec) for 15 min at 4 °C (20 μl per 10^7^ cells) in MWB. Excess antibody was again removed by washing with HBSS (−/−) and cells were magnetically sorted following the manufacturer's instructions (Milteny Biotech, Surrey, UK). Cells were eluted with OPC media (DMEM F12, 2 mm sodium pyruvate, 60 μg N-acetyl-cysteine (Sigma-Aldrich), 5 μg/ml Insulin (Gibco), 21 mmd-Glucose (Sigma-Aldrich), 50 μg/ml apo-transferrin (Sigma-Aldrich), 16.1 μg/ml putrescine (Sigma-Aldrich), 40 ng/ml sodium-selenite (Sigma-Aldrich) and 60 ng/ml progesterone (Sigma-Aldrich). Upon OPC removal cells were incubated with 2 μl of Biotin-MOG antibody per 10^7^cells (R and D systems, Abingdon, UK) for 25min at 4 °C in 500 μl MWB to label oligodendrocytes. Excess antibody was washed with HBSS (−/−) and cells were incubated with secondary IgM-microbeads (Miltenyi Biotec) for 15 min at 4 °C (20 μl per 10^7^ cells) in MWB. Excess antibody was again removed by washing with HBSS (−/−) and cells were magnetically sorted following the manufacturer's instructions (Miltenyi Biotech). Oligodendrocytes were also eluted with OPC media.

### Proteome Sample Preparation

##### Experimental Design and Statistical Rationale

OPCs isolated from neonatal (P0-P2), 3–4 months old and 15–18 months old rats were processed as depicted below. Each biological replicate and condition consisted of cells isolated from independent rats. A sample size of six biological replicates was used for each age (neonatal, young, and aged OPCs) providing us with sufficient numbers to accommodate variability. The samples were labeled using TMT 10 plex (ThermoFisher Scientific, Altrincham, UK) in which each sample was labeled with a different isotype to allow the distinction of the proteome of each sample. The 6 biological sample were divided into two different TMT 10 plexes (named as multiplex 1 and 2) to enable labeling of all the samples avoiding a potential plate effect and to provide relative quantitation across the three ages. Multiplex 1 included neonatal 1–3, young 1–3 and aged 1–3 sample, whereas multiplex 2 included neonatal 4–6, young 4–6 and aged 4–6. A sample consisting of pooled material from all replicates and conditions was labeled with the 10^th^ TMT tag with the intent of minimizing the number of missing values between multiplexes but was not used for statistical analysis. Furthermore, each multiplex containing three biological replicates of each age was pre-fractionated as describe below and each fraction ran twice in different rounds of LC-MS to increase the proteome coverage. We used LIMMA (linear model for microarray analysis) (23) statistical analysis because of its robustness in dealing with the number of missing values and variability encountered on high-throughput quantitative proteomic studies of primary cells as ours. Neonates were used as control condition except when comparing young *versus* aged rats where young was the control.

##### Sample digestion and TMT labeling

Cells obtained by magnetic cell sorting were pelleted by centrifugation and supernatant was removed. The cell pellet was lysed using lysis buffer (8 m urea, 100 mm triethylammonium bicarbonate [TEAB] pH 8.0) (Life-Technologies, Altrincham, UK) followed by freeze-thawing in dry ice and ultrasonic bath incubation. Each lysate was immediately reduced with 20 mm dithiothreitol (DTT) (Sigma-Aldrich) in TEAB at RT for 60 min, alkylated with 40 mm iodoacetamide (Sigma-Aldrich) in the dark for 60 min at room temperature (RT) and digested overnight at RT with 1 μg endoproteinase Lys-C (Promega, Madison, Winsconsin). The following day, the solution was diluted to a final urea concentration of 1 m and 1 μg of modified trypsin (Promega) per 100 μg of cell lysate was added and incubated for 3 h at RT. The samples were acidified with trifuoroacetic acid (TFA) (Life Technologies) (0.1% (v/v) final concentration) and debris were pelleted by centrifugation at 15,000 × *g* for 10 min and supernatant frozen at −80 °C until peptide concentration and clean-up.

PorosOligo R3 (Life-Technologies) resin was equilibrated in 0.1% TFA. Each sample was desalted by sequentially incubating for 5 min with occasional vortexing with 20 μl of slurry (about 10 μl resin). The samples were then spun down, and the supernatant transferred to a fresh microtube with another 20 μl slurry. This step was repeated 3 times. Peptide-loaded resins were combined and packed into p200 tips over a 3 m Empore C8 “plug” (about 1 cm long), washed twice with 50 μl 0.1% TFA and eluted with 200 μl 60% acetonitrile (ACN) (adapted from Rappsilber *et al.* 2003 (21)) and quantified using Qubit^TM^ (Life Technologies). The volume equivalent to 12 μg of peptides (equivalent to the amount of peptide in the lowest yielding condition) were transferred to new microtube to be dried down before TMT-labeling.

Samples were labeled according to manufacturer's instructions (ThermoFisher Scientific) with minor modifications. Briefly, each dry desalted sample was resuspended in 122.5 μl 100 mm TEAB and each TMT label (0.8 mg) resuspended and mixed in 41 μl of pure acetonitrile, then mixed for labeling. Samples were labeled for one hour at RT, quenched with 5% hydroxylamine for 15 min and multiplexed. Each multiplex was then dried and stored at −80 °C for pre-fractionation for total proteome analysis.

##### Alkaline Reverse-phase Prefractionation and LC-MS/MS Analysis

TMT-10plex labeled peptides from OPCs were combined and fractionated using the alkaline reversed-phase chromatography (22) on Dionex Ultimate RSLC system. Separation was carried out on an XBridge BEH C18 column (2.1 × 100 mm, 5 μm, 130 Å) (Waters) using a gradient of acetonitrile (2 to 98%) in 10 mm TEAB with a flow rate of 300 μl/min and a running buffer pH = 10. Ninety-six fractions were collected and concatenated into 18 fractions (supplemental Fig. S1) and concentrated to dryness using a speedvac with a chilled vacuum trap and stored at −80 °C.

Eighteen fractionated peptide samples were analyzed in replicate on an Orbitrap Fusion Tribrid (Thermo Scientific) mass spectrometer interfaced with Dionex Ultimate 3000 nanoRSLC system. Nanospray LC system comprised of a 20 mm enrichment column (ReproSil-Pur 120 C18-AQ, 7 μm, Dr. Maish, Ammerbuch-Entringen, Germany) and a Picofrit (New Objective) analytical column (ReproSil-Pur 120 C18-AQ, 2.4 μm, 50 cm length). Peptides were separated using a gradient of acetonitrile with 0.1% formic acid up to 80% over a period of 110 min. TMT labeled peptides were analyzed using the following data dependent acquisition parameters: Scan range for full MS, *m*/*z* 400–1600, resolution 120,000 (200 *m*/*z*), charge state(s) 2–7, higher collision dissociation fragmentation (The fragmentation mode was a MS/MS fragmentation of top 15 most intense peptide ion peaks fragmented through higher energy collisional dissociation (HDC) with 35% energy) at 60,000 resolution, precursor isolation window *m*/*z* 1.3 with *m*/z 0.3 offset. Fragmented peptides were excluded for 30 s, monoisotopic selection precursor (MIPS) was enabled with constant internal calibration using fluoranthene ion.

Mass spectrometry data were analyzed using Proteome Discoverer 2.1 (PD) (Thermo Fisher Scientific) software with search engines Mascot v2.6.0, Amandav2.1.5.4882 and Sequest HTv.1.1.0.158 nodes. Data was searched using latest Uniprot *Rattus norvegicus* protein database with common laboratory contaminants included (2016_03 version with 30030 entries including *Rattus norvegicus* and common laboratory contaminant sequences). Search parameters included 2 missed cleavage sites, oxidation (M) and deamidation (N, Q) as variable modifications. Tandem mass tag (229.163Da) at N terminus and lysine residue and carbamidomethylation on cysteine residue were set as fixed modifications. The mass tolerances on precursor and fragment masses were set at 10 ppm and 0.05 Da, respectively. False discovery rate (FDR) cut-off value was set at 0.01. Unique peptide spectrum matches with protein information and non-normalized reporter ion intensity was exported and used for statistical analysis.

##### Statistical Analysis of TMT-labeled Samples

Statistical analysis of TMT labeled samples was performed with RStudio® software. The data from the time course analysis consisted of exported peptide-to-spectrum matches (PSMs) from two multiplexes containing three biological replicates each (a total of 6 biological replicates). Identification results from technical replicates and/or fractions were merged and any repeated protein groups were removed. PSMs having ≥50% isolation interference were removed. PSMs from proteins from the common contaminant database were removed. The false discovery rate (FDR) for PSM assignments was calculated against a decoy database using Percolator v2.05 node. PSMs with missing value in one or more conditions within a biological replicate were removed. The intensity values of each sample and each TMT label were log_2_-transformed and then subjected to median sweeping on the log_2_ intensity data by each plate which provides a protein abundance value for further LIMMA (Linear Model for Microarray Analysis) statistical analysis (23).

LIMMA analysis provides sufficient power to deal with low replicate numbers and additional missing values (24). We therefore carried out unpaired LIMMA analysis comparing all and corrected them for multiple testing using the R function provided within VSClust (25). All proteins with q-values below 0.05 (5% FDR) and log_2_ change over 0.6 (more than 1.5-fold change) when compared with neonates were considered to be regulated. In the case of young adult and aged OPC comparison, all proteins with q-values below 0.05 (5% FDR) and log_2_ change over 0.6 (more than 1.5-fold change) were considered significantly regulated.

##### Downstream Bioinformatics Analysis

All protein identification data were mapped to gene symbols using UniProt version 2016_03 (26). Annotation for Gene Ontology (GO) pathways such as biological processes, molecular function, cellular compartment as well as Kyoto Encyclopedia of Genes and Genomes (KEGG) pathways was performed using DAVID (27). When analyzing the GO terms of the total protein groups detected, the whole proteome included in DAVID for *Rattus norvegicus* was used as background using Benjamini-Hochberg (28). The statistical test PCA clustering analysis was performed using the Log_2_ transformed protein intensity. For the cluster analysis, we calculated the log_2_ protein intensity mean over all biological replicate values for each condition of those proteins with q-value < 0.05 and log_2_ change over 0.6 (more than 1.5 fold change) when compared neonatal OPCs with young and aged OPCs. Fuzzy c-means clustering (29) was applied using the “FcmClusterPEst.R” R function (https://bitbucket.org/veitveit/vsclust/src/master/ (25)) and “Mfuzz” R library. We determined the value of the fuzzifier and obtained the number of clusters according to Schwämmle and Jensen (30) (supplemental Table S3). The GO term analysis for the different clusters obtained with Fuzzy c-means clustering as well as for the protein groups with more than 1.5-fold change expression between young and aged OPCs were obtained with DAVID, using the total protein groups detected in our proteome as background. For the heatmap, the Z score from each age category was used. The Z score was calculated using the scale function within RStudio® software. The heatmaps were calculated using the “pheatmap” R package from Kolde (https://cran.r-project.org/web/packages/pheatmap/) from the R Bioconductor tool set and the protein groups shown in each heatmap were obtained from the corresponding DAVID GO term shown in the tittle of each heatmap. To analyze GO terms of the differentially expressed proteins in the different aged group, each list was uploaded to DAVID and compared with the total proteome detected in the Ageing OPC proteome. Then, to elaborate the list of proteins involved in each of the GO terms, the Uniprot accession protein list provided by DAVID was used. The GO terms and the Uniprot accession protein lists associated to each GO term are provided in the supplemental Table S5 and the fold enrichment of the GO terms that were significantly enriched in the category Biological processes (supplemental Fig. S3), KEGG pathway (supplemental Fig. S4), Cellular component (supplemental Fig. S5), or Molecular function (supplemental Fig. S6) in each of the clusters was represented using a heatmap value for the fold enrichment compared with the total proteome, which was used as background. The GO term network for proteins differentially regulated between young and aged proteins was created using the Cytoscape application BINGO (31). In supplemental Fig. S7, the different GO biological processes that are associated are linked with a gray line. The size of the node indicates the number of proteins among the regulated proteins that are involved in that GO term. The bigger the node the higher the number or proteins. Each node is then divided in 2 colors, blue and orange. Blue indicates proteins that are downregulated in aged OPCs and orange proteins that are up-regulated in aged OPCs. Again, the size of each color is associated to the number of proteins.

The correlation coefficient between RNA expression from young and aged OPCs and the proteome of young and aged OPCs was performed as follows: RNAseq data (GSE134765) was pre-processed through sortMeRNA (Kopylova *et al.*, 2012) to filter out ribosomal RNA reads, adapter was trimmed and quality paired reads were extracted using Trimmomatics (32). Rat cDNA (Rnor_6.0) was indexed and reads were quantified using Salmon quasi mode (33). Salmon output was processed through tximport (34) and Deseq2 (35) and normalized length scaled TPM counts were calculated. Normalized log_2_ transformed values of transcript levels were used for correlation analysis with log_2_ transformed proteomic data set (15).

Ensemble gene IDs corresponding to Uniport protein IDs were extracted. Genes for which expression value was not determined ('NA') in any one sample were removed. Finally, 6110 genes for 'young' OPC samples and 6101 genes for ‘aged’ OPC samples remained. For each gene, a correlation coefficient (Biweight midcorrelation or bicor) between transcript levels and protein levels was estimated and the corresponding Student *p* value was determined using bicorAndPvalue function (36) in “R.” The advantage of bicor over Pearson's correlation coefficient is based on its robust measurement in presence of outliers. *p* value was adjusted using FDR (Benjamini-Hochberg) and the adjusted *p* value <0.05 was considered significant.

##### Western Blot Analysis

Protein lysate was mixed with NuPAGE loading buffer and NuPAGE reducing agent (Life Technologies) and boiled for 10 min at 95 °C. Five μg of protein lysate were loaded into Bolts 4–12% gels (LifeTechnologies). Electrophoresis gel was run at 120 volts (V) for 90 min and then gels were transferred onto a methanol pre-activated PVDF membrane (Millipore). The transfer was done using 1X Bio-Rad transfer buffer with 20% of ethanol at 100V for 90 min. Upon transfer the membranes were blocked with Blocking buffer (TBS blocking agent 1:1 (Li-Cor, Lincoln, Nebraska) with TBS (Fischer Scientific)-0.1% Tween (Sigma-Aldrich)) for 1 h at RT. Membranes were incubated overnight at 4 °C with the different antibodies (Rabbit MOBP (Abbexa, Cambridge, UK, abx002902) 1:500; Rat MBP (Serotec, Kidlington, UK, MCA490S) 1:500; Mouse PADI2 (Proteintech, Manchester, UK, 66386–1-lg) 1:500; Goat FABP5 (Cell Signaling Technologies, Leiden, Netherlands, 39926) 1:500; Mouse CNPase (Sigma-Aldrich, C5922) 1:500; Goat MOG (R and D systems, AF2439) 1:1000; Rabbit PLP (Abcam, Cambridge, UK, ab28486) 1:500; Mouse CRYAB (Abcam, ab13496) 1:500; Rabbit proteasome 19S (Abcam, ab137109), alpha tubulin (1:1000) (Millipore, T9026). Following antibody incubation membranes were washed with TBS-0.1% Tween (TBST) three times for 10 min. Membranes were then incubated with fluorophore conjugated secondary antibodies (Li-Cor) for 2 h at RT and developed using Li-Cor developing machine. Western blot analysis was performed measuring the integrated density through the Li-Cor software and normalized by the signal of HRP conjugated β-actin (Sigma-Aldrich, Birmingham, Alabama). Statistical analysis was performed with GraphPad Prism version 8.0. First, normality was tested and if the number of samples was too low, normality test using residuals was performed. If samples were normally distributed 1 way, ANOVA with Sidak's multiple comparisons test was performed and if samples were not normally distributed samples were analyzed using Kruskal Wallis and Dunn's Multiple Comparison Tests. Statistics were represented as follows: ns (not significant), *<*p* < 0.05; **p < 0.01, ***p < 0.001 and ****p < 0.0001.

##### Immunocytochemistry

Cells were plated on poly-d-lysine coated coverslips at a density of 15,000 cells/9 mm coverslips for adults and 2,000 cells/9 mm coverslip in the case of neonates. Cell were cultured with OPC media supplemented with 1 ng/ml PDGF-aa and 1 ng/ml b-FGF in the case of OPCs. After 6 days *in vitro* cells were fixed with 4% PFA for 10 min and blocked with 5% normal donkey serum (NDS) (Sigma-Aldrich) with 0.01% Triton X-100 (Sigma-Aldrich) in PBS for 1h at RT. Cells were incubated for 1 h at RT with the corresponding antibodies (Goat-anti MOBP (Abbexa, abx002902)1:500; Mouse PADI2 (Proteintech, 66386–1-lg) 1:500; Goat FABP5 (Cell Signaling Technologies, 39926) 1:500; Mouse CRYAB (Abcam, ab13496) (1:500), Rabbit anti-Claudin 11 (Abcam, ab53041) 1:500, Goat anti-MMOG (R and D systems, AF2439) 1:500) in 5% NDS with 0.01% Triton-X-100 (Sigma-Aldrich). Following washing, fluorescently labeled secondary antibodies (Life Technologies) were used in a 1:500 dilution in 5% NDS with 0.01% Triton-X for 1 h at RT. Anti-A2B5 antibody (Millipore, MAB312, 1:500) was incubated sequentially to the secondary antibody incubation to avoid cross-reactivity. Nuclei were stained with Hoechst (2 μg/ml) (Sigma-Aldrich) for 5 min followed by two washes with PBS prior to mounting the coverslips with Fluoromount G (Southern Biotech, Birmingham, Alabama). Statistical analysis was performed with GraphPad Prism version 8.0. First, normality was tested and if the number of samples was too low normality test using residuals was performed. If samples were normally distributed 1 way, ANOVA with Sidak's multiple comparisons test was performed and if samples were not normally distributed samples were analyzed using Kruskal Wallis and Dunn's Multiple Comparison Tests. Statistics were represented as follows: ns (not significant), *<*p* < 0.05; **p < 0.01, ***p < 0.001 and ****p < 0.0001.

##### Flow Cytometric Analysis

Cells were isolated by magnetic cell sorting. The positive fraction was subjected to staining as follows: 10^6^ cells were collected in media, spun down at 350 × *g* for 5 min and washed once with PBS. Cells were resuspended in 1:100 Zombie Violet (Biolegend, London, UK, 423113) and incubated for 15 min at RT in the dark. Cells were then topped up with PBS1X and pelleted by centrifugation. Cell pellets were re-suspended and incubated in 10% fetal calf serum in PBS for blocking. Then cells were pelleted and re-suspended in primary antibodies for 30 min except for A2B5-PE (Miltenyi, 130–093-581), which was added in the last 10 min of the incubation. Cells were washed with PBS - 0.5% BSA and pelleted by centrifugation. Cell pellets were re-suspended in the appropriate secondary antibodies solution in 5% fetal calf serum in PBS, incubated 15 min at RT and then washed with PBS-0.5% BSA, centrifuged and resuspended in 0.5 ml “Flow media” (2 mm sodium pyruvate (Life Technologies), 4% SOS (Cell Guidance Systems, Cambridge, UK) and 10 μg/ml insulin (Gibco) in Hibernate A). The primary antibodies used were; anti- CD11B/C PerCP-Cy5.5 Clone OX-42(Biolegend, 201819), anti-rat erythroid cells Clone OX83 (Biologend, 250402), A2B5-PE (Miltenyi Biotec, 130-093-581) and goat-MOG (R and D Systems, AF2439). The secondary antibodies used include anti-goat Alexa Fluor 647 (Life Technologies) and Pe-Cy7 (Biolegend). Statistical analysis was performed with GraphPad Prism version 8.0 for A2B5^+^MOG^−^ population as that is our population of interest. Because of working with percentages, samples were analyzed using Kruskal Wallis and Dunn's Multiple Comparison Tests. Statistics were represented as follows: ns (not significant), *<*p* < 0.05; **p < 0.01, ***p < 0.001 and ****p < 0.0001.

##### Immunohistochemistry

Rats were terminally anesthetized and fixed by intracardiac perfusion using 4% (w/v) PFA. Brains were removed, post-fixed in 4% (w/v) PFA overnight at 4 °C, cryoprotected with 20% (w/v) sucrose for 24–48 h, embedded and frozen in OCT medium, and stored at −80 °C. Tissues were sectioned at 12 μm and collected onto poly-d-lysine-coated glass slides. 12 μm cryo-sections were dried for 1 h at RT and then rehydrated in PBS. After rehydration, slides were washed 3 × 5 min with TBS-0.25% Tween-20 (Sigma-Aldrich). Sections were then exposed to 1X Citrate Buffer pH 6.0 at 90C for 5min and then the sections were let to cool down at room temperature for 30 min. Sections were then washed 3 × 5 min with TBS-0.25% Tween-20 (Sigma-Aldrich). After wash sections were permeabilized for 30min with 1% Triton X-100 (Sigma-Aldrich) and then blocked with 10% NDS and 0.25% Tween-20 for 1 h at RT. After blocking, slides were incubated with primary antibodies in 1% NDS in TBS-0.25% Tween-20 (Sigma-Aldrich) overnight at 4°C (Rabbit anti-OLIG2, 1:500 (AB9610, Millipore); Goat anti-PDGFRα (R and D systems, AF1062); Rabbit anti-proteasome 20S (Abcam, ab22673) (1:200) or Rabbit anti-PFDN5 (Abcam, ab129116)(1:200). Slides were then incubated with the appropriate Alexa Fluor® secondary antibodies 1:500 (Life Technologies) for 2h at RT. Nuclei were stained with Hoechst (2 μg/ml, Sigma-Aldrich) for 5 min at RT, before the coverslips were mounted using Prolong Gold (Life-technologies). Per-cell intensity measurements in immunofluorescent sections were quantified using *CellProfiler (v3.1.8*) and *CellProfiler Analyst* software. First, Hoechst+ nuclei were detected using the *CellProfiler* “IdentifyPrimaryObjects” module and various intensity features were measured within the PDGFRα channel from the region surrounding each nucleus. PDGFRα+ cells were distinguished from PDGFRα- cells within *CellProfiler Analyst*, using a supervised learning approach. The PDGFRα+ cells were then identified within *CellProfiler*, and the mean intensity within the local area was measured for each cell in the channel of interest (PFDN5 or 20S proteasome).

## RESULTS

##### Generating Proteomes of Acutely Isolated OPCs

**Fig. 1:**
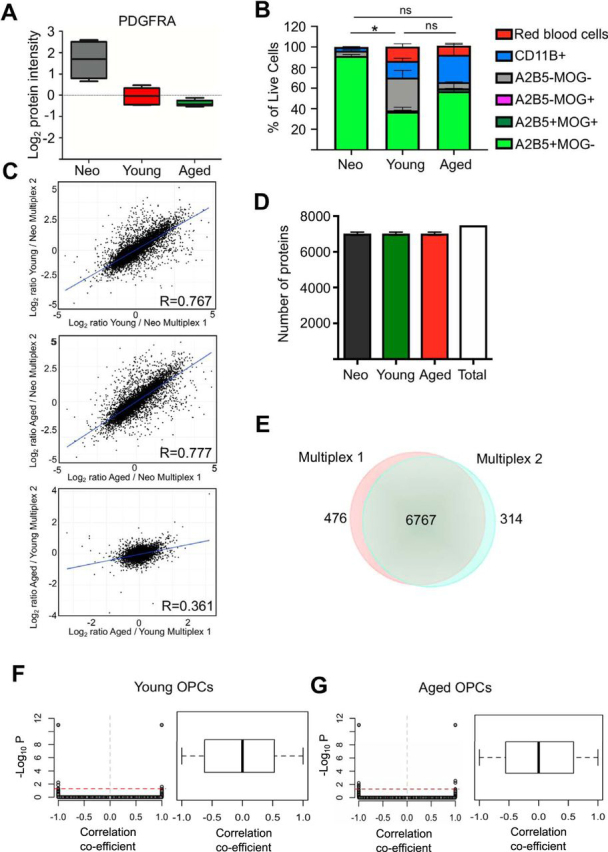
**Validation of OPC isolation method and overview of the ageing OPC proteome.***A*, Boxplot showing the log_2_ protein intensity for PDGFRA in neonate, young and aged OPCs (*n* = 6). *B*, Graph showing flow cytometric analysis of purity of freshly isolated OPCs at the three different ages shown as a percentage of live cells (*n* = 4, Mean ± S.E.M. shown, statistics shown for A2B5^+^MOG, Kruskal-Wallis *p* = 0.038*, Dunn's Multiple test comparison between neonate and young *p* = 0.0172, neonate and old *p* = 0.34 and young and old *p* = 0.60). *C*, Scatter plot showing the correlation of the log_2_ changes of all protein groups among the 2 multiplexes. *D*, Graph showing the number of protein groups detected per age group. A total of 7481 protein groups were detected with 1% FDR. *E*, Venn diagram of the proteins identified with high confidence in multiplex. *F*, *G*, Graphs showing the relationship between protein level and transcript level for young (*F*) and aged (*G*) OPCs. Scatter plots show the representation of the correlation coefficients (Biweight midcorrelation coefficient) and the corresponding adjusted *p* values of significance (*p* values were plotted as negative log_10_. Threshold (red dotted line) corresponds to FDR adjusted *p* value 0.05). Boxplots show the distribution of correlation coefficients for each detected protein.

To assess differences in the proteome of OPCs at different ages, we isolated OPCs using magnetic cell sorting to select for A2B5+ cells from neonatal (P2), young (3–4 months old) and aged (15–18 months old) female rats based on the higher prevalence of MS in women than in men (37). An antibody against A2B5 was used for OPC selection because i) the high yield and consistency of isolation of adult OPCs (1 million cells per adult brain) and ii) the age-associated decrease in expression of other OPC markers such as PDGFRA (Fig. 1*A*). The three ages were selected for the following reasons: (1) developmental myelination occurs during neonatal stages, and OPCs isolated during this period have been extensively studied *in vitro* (38, 39, 40); (2) 3–4 months is the adult rodent age where remyelination is highly efficient (5); and (3) 15–18 months old is the age at which remyelination is already impaired and is also slightly over half of the life-span of rats in captivity. Therefore, this can be regarded as close to the age at which most MS patients generally transition from relapsing-remitting to progressive MS (5, 41). We termed the OPCs in each group as neonatal, young, and aged OPCs, reflecting the age of the animal from which they were isolated rather than making any inference about the age of individual isolated cells.

To determine the quality of the OPC isolation, we compared the purity of the samples isolated in each age group using a panel of antibodies to detect erythroid cells (Clone OX83), microglia (CD11b), oligodendrocytes (MOG) and OPCs (A2B5). Although in neonates over 90% of the live cells were A2B5^+^ cells with little microglia or red blood cells, in adults (young and aged OPCs) the samples were contaminated with 26% of CD11b^+^ microglia, 2% of A2B5^+^MOG^+^ early differentiated oligodendrocytes, 0.4% of A2B5-MOG^+^ oligodendrocytes and 15% of red blood cells (Fig. 1*B*).

To determine the differences between the neonatal, young and aged OPC proteome we analyzed six animals (each treated as an independent biological replicate) for each age group. Cells were lysed immediately after isolation and lysates were subjected to two-step Lys-C/trypsin digestions and resulting peptides were labeled with TMT-10plex and combined (multiplexed), where peptides coming from each animal were marked with a different mass tag. The resulting multiplexed peptides were fractionated and concatenated into 18 fractions (supplemental Fig. S1A) and subjected to liquid chromatography-tandem mass spectrometry (LC-MS/MS) (22). The six animals from each of the 3 age groups were divided equally into two different multiplexed LC-MS/MS analyses. These two multiplexes showed a high correlation in the log_2_ changes between young and neonatal or aged and neonatal OPC samples, whereas this correlation was significantly lower in the log_2_ changes between aged and young OPCs. These data suggest there was no batch effect in the analysis and the decreased correlation between the log_2_ change ration between aged and young OPCs in multiplex 1 and 2 reflects age-associated intrinsic variability of primary cells (Fig. 1*C*). The peptide spectra were analyzed using Proteome Discoverer 2.1 and identified searching against UniProt *Rattus norvegicus* version 2016_03 database (26) with maximum threshold of 1% false discovery rate (FDR) at protein and peptide level. Quantitation of relative protein expression changes was based on the signal of the TMT tags used to label the OPCs arising from each animal/age. We only accepted protein groups with at least two high confidence peptides identified, from which at least one was unique to that protein. We detected 7480 protein groups (supplemental Table S1) (Fig. 1*D*), with an average of 11 peptides per protein and an average coverage per protein of 25%. From the total proteins, 6767 were detected in both plates, whereas 476 were detected only in plate 1 and 314 in plate 2 (Fig. 1*E*). We analyzed the tissue enrichment of the whole proteome, which covered a large portion of brain specific proteins (28) (adjusted *p* value 5.3 × 10^−14^) (Fig. S1B). Although protein and RNA often show a poor correlation with each other, with a high degree of variability of the correlation coefficient depending on the protein (17), this relationship has not been extensively examined in OPCs. Thus, we performed a correlation analysis between the proteome and RNA transcript levels in young and aged OPCs (15). This analysis revealed, a low linear correlation between transcripts and proteins. We obtained a correlation coefficient between −0.5 and 0.5 in most cases, however the statistical significance was below the FDR threshold (0.05) (Fig. 1*F*, 1*G*).

**Fig. 2:**
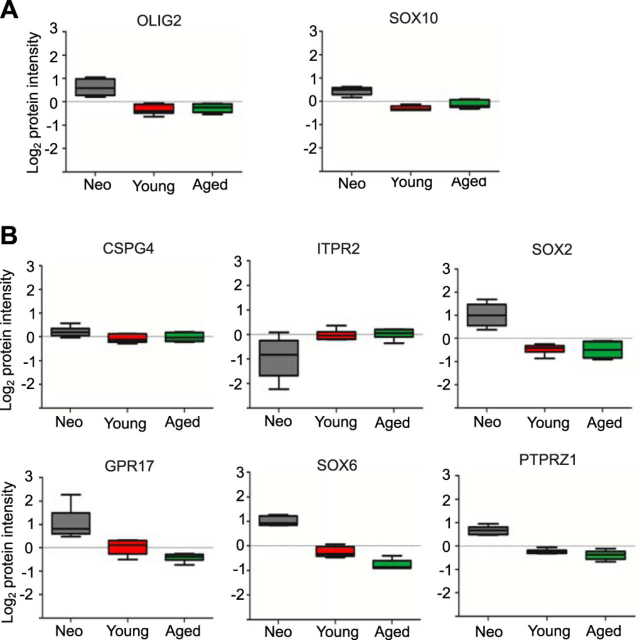
**Expression of OPC cell markers.***A*, Boxplots displaying the log_2_ protein intensity for oligodendrocyte lineage cell markers OLIG2 and SOX10 in neonate, young and aged OPCs (*n* = 6). *B*, Boxplots showing the log_2_ protein intensity of various OPC specific markers in the three age groups (*n* = 6).

We then assessed the expression of oligodendrocyte lineage markers looking at the log_2_ protein intensity, accomplished by plotting the reporter intensities for each condition after normalization and peptide to protein aggregation based on median values. Oligodendrocyte lineage cell markers such as OLIG2 and SOX10 (Fig. 2*A*), as well as OPC markers PDGFRA, CSPG4, SOX2, and GPR17, were expressed in all three groups: neonatal, young and aged OPCs (Fig. 1*A*, 2*B*). The expression of some of these markers decreased with ageing, corroborating mRNA expression (2). In contrast, the recently identified OPC marker ITPR2 (42) as well as CSPG4, which is expressed by other CNS resident cell types such as perivascular cells (43, 44) and ageing/reactive microglia (45, 46), had stable expression across the age groups.

**Fig. 3:**
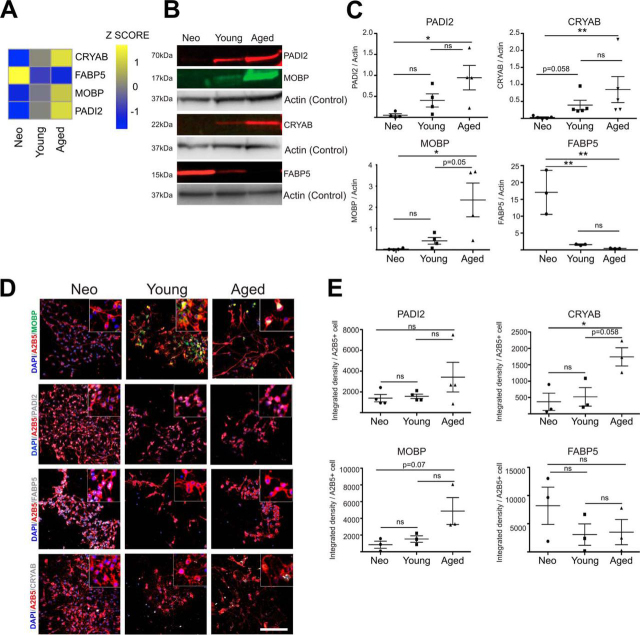
**Validation of the ageing proteome.***A*, Heatmap showing the Z score expression of 4 randomly chosen proteins. Z score was calculated and heatmaps indicates the mean Z score for the six independent biological replicates of the proteomics dataset. Lower expression is represented in blue, and higher expression is shown in yellow. *B*, Western blotting images validating the proteome expression analysis in acutely isolated OPCs (*n* = 3–6, Mean ± S.E.M. shown) *C*, Graphs show the densitometry quantification for PADI2, CRYAB, MOBP and FABP (*n* = 3–4). *D*, *E*, Immunocytochemistry validation and quantification of the intensity of selected proteins per A2B5^+^ cell after 7 DIV. Scale bar 100 μm (*n* = 3, Mean ± S.E.M. shown).

After examining for the expression of OPC markers, we next validated the accuracy of the expression changes detected. Four proteins that significantly change expression with ageing were selected - MOBP, PADI2, CRYAB, and FABP5, and their expression in each age group was assessed by Western blotting (Fig. 3*A*–3*C*). MOBP (1-way ANOVA *p* = 0.0144*, Sidak's Multiple comparison test neonatal *versus* young *p* = 0.92, neonatal *versus* aged *p* = 0.0199*, young *versus* aged *p* = 0.0506), PADI2 (1-way ANOVA *p* = 0.0281*, Sidak's Multiple comparison test neonatal *versus* young *p* = 0.54, neonatal *versus* aged *p* = 0.0284*, young *versus* aged *p* = 0.21) and CRYAB Kruskal-Wallis *p* = 0.0012*, Dunn's Multiple comparison test neonatal *versus* young *p* = 0.0589, neonatal *versus* aged *p* = 0.0089**, young *versus* aged *p* = 0.99) are significantly enriched with ageing being statistically significant especially when comparing neonatal and aged OPCs. FABP5 on the other hand, significantly decreases its expression with ageing similar to what was observed in the proteome (1-way ANOVA *p* = 0.0028**, Sidak's Multiple comparison test neonatal *versus* young *p* = 0.007**, neonatal *versus* aged *p* = 0.0049**, young *versus* aged *p* = 0.979) (Fig. 3*C*). Similarly, the expression of each protein per A2B5^+^ cell was also assessed by immunohistochemistry, where the proteins show similar trends to the proteome (Fig. 3*D* and 3*E*, supplemental Fig. S2). CRYAB shows a statistically significant increase (1-way ANOVA *p* = 0.0238*, Sidak's Multiple comparison test neonatal *versus* young *p* = 0.9760, neonatal *versus* aged *p* = 0.0372*, young *versus* aged *p* = 0.0598) whereas PADI2 (1-way ANOVA *p* = 0.4194), MOBP (1-way ANOVA *p* = 0.0565) and FABP5 (1-way ANOVA *p* = 0.3555) show nonsignificant trends (Fig. 3*E*). These results corroborated the abundant changes obtained by high-throughput proteomic analysis.

##### The Neonatal OPC Proteome Is Distinct from That of Adult Counterparts

**Fig. 4:**
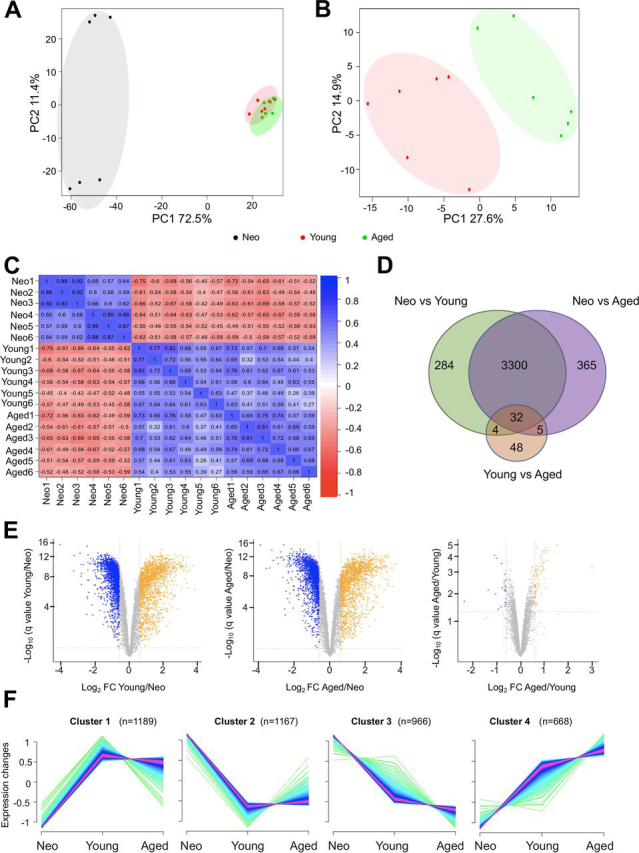
**The neonatal OPC proteome is significantly different to the adult OPC proteome.***A*, Principal component analysis (PCA) plot showing the segregation of neonate, young and aged OPC proteomes on components 1 and 2. *B*, PCA plot showing the segregation of young and aged OPC proteomes on components 1 and 2. *C*, Correlation matrix between the biological replicates within and across the age groups. Neonatal OPCs show a negative correlation with adult OPCs. *D*, Venn diagram of the number of common and different proteins showing a significant expression change between neonatal *versus* young, neonatal *versus* aged, and young *versus* aged OPCs. *E*, Volcano plots illustrating differential expression of proteins between neonatal, young and aged OPCs. Colored proteins are significantly regulated with FDR < 0.05. Blue in indicates a Log_2_ change < (-0.6) (fold change < (-1.5)) whereas orange refers to proteins with Log_2_ change > 0.6 (fold change > 1.5). *F*, Unsupervised fuzzy C-means clustering showing the protein dynamics of those proteins with FDR < 0.05 and more than 1.5 fold change expression when compared with neonatal OPCs with *n* representing the number of proteins per cluster.

To better understand how the OPC proteome changes with ageing, principal component analysis (PCA) was performed based on the log_2_ protein intensity. PCA analysis showed that neonatal OPCs cluster separately from young and aged OPCs. The distinction between young and aged OPCs was not clear, because of neonatal OPCs dominating the first (PC1) and second principal components (PC2) (Fig. 4*A*). However, when PCA analysis was performed with only young and aged OPCs, they were clearly discriminated from each other (Fig. 4*B*). These results were also observed when analyzing the correlation of the biological replicates. There was a positive correlation between the replicates within each age group and also between the proteome of young and aged OPCs, whereas the neonatal OPC proteome correlated negatively with young and aged OPC proteome (Fig. 4*C*).

We next investigated the protein expression differences between the three age groups. To determine which of these expression changes was significant, unpaired LIMMA (linear model for microarray analysis) analysis was performed (23). Taking into account the differences in purity between neonatal and adult OPCs (young and aged) (Fig. 1*B*), a high stringency analysis was applied: only those proteins present in at least 5 out the 6 biological replicates were considered for statistical analysis (6842 proteins out of the 7481 total proteins) and of these, only proteins having a q value below 0.05 (5% FDR) and fold change over 1.5 (Log_2_ change over 0.6), were regarded as significantly regulated. We detected 3620 proteins significantly regulated between neonatal and young OPCs, 3702 between neonatal and aged OPCs, whereas only 89 proteins were differentially expressed between young and aged OPCs. (Fig. 4*D*, 4*E*; supplemental Table S2).

**Fig. 5:**
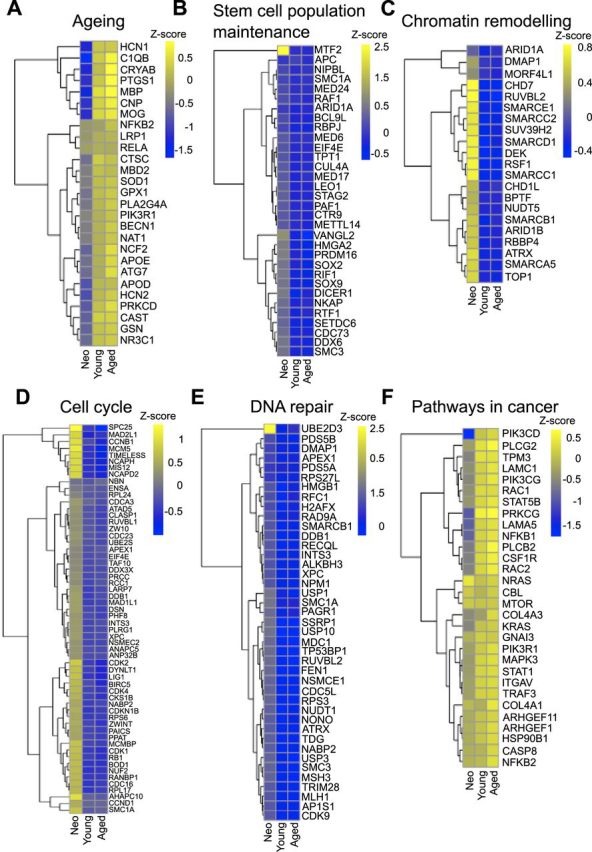
**OPCs show classic ageing hallmarks.** Heat maps showing the mean Z score per age group for proteins associated with ageing (*A*), stem cell maintenance (*B*), chromatin remodelling (*C*), cell cycle (*D*), DNA repair (*E*) and cancer pathways (*F*). Row Z score indicates the mean of six independent biological replicates and lower expression is represented in blue, and higher expression is in yellow.

To characterize how proteins change their expression from neonate to young and aged OPCs, these were first grouped using unsupervised fuzzy c-means clustering (29) (supplemental Table S3). This unsupervised clustering classified the protein groups into four distinct clusters according to their expression profile. Cluster 1 (*n* = 1189) and Cluster 4 (*n* = 668) included proteins that increased their expression with ageing, whereas Cluster 2 (*n* = 1167) and Cluster 3 (*n* = 966) included proteins that were decreased with ageing (Fig. 4*F*). We then used GO analysis to determine whether these profile clusters encompassed distinct biological processes. As expected, ageing was one of the most enriched GO Biological processes in cluster 4 (Fig. 5*A*). In addition, we also observed many of the hallmarks of ageing (47), including decreased levels of proteins involved in the maintenance of stem cell populations (*e.g.* SOX2 and SOX9) (Fig. 5*B*), chromatin remodelling (*e.g.* CHD7and ARID1) (Fig. 5*C*), cell cycle control (*e.g.* CDK1 and CDK2) (Fig. 5*D*) and in DNA repair (*e.g.* H2AFX or NUDT1) (Fig. 5*E*), as well as an increase in expression of proteins involved in cancer pathways (*e.g.* NRAS or KRAS) (Fig. 5*F*).

Cluster 1 includes proteins that increase their expression once development is completed with little change during adulthood. This cluster contains proteins involved in oxidative phosphorylation, neurotransmitter secretion and metabolism. It also includes proteins associated with the classical neurodegenerative diseases, Alzheimer's disease (AD) such as MAPT or NOS1, Parkinson's disease (PD) like SNCA or cytochrome *c* oxidase complex proteins and Huntington's disease (HD) such as HAP1. Cluster 4 comprised proteins that increase in expression from development and through ageing. This cluster includes proteins associated with immune responses, endocytosis, regulation of actin cytoskeleton, and autophagy, and are located mainly in the plasma membrane, exosome, and lysosome. Cluster 2 consists of proteins that are decreased after development and remain similar through adulthood and includes proteins involved in mRNA splicing, transcription, translation, and DNA repair, which are located mainly in the nucleoplasm, nucleolus, spliceosomal complex and ribosome subunits. Cluster 3 includes proteins that decrease with ageing. These are proteins involved in DNA replication, cell division, brain development and stem cell population maintenance and are located mainly in the nucleus and chromatin. The complete GO annotation and KEGG pathway analysis for each cluster is represented using heatmaps in supplemental Fig. S3, S4, S5, S6 and supplemental Table S4. In the heatmap, annotation terms significantly enriched in each cluster when compared with the total proteome are represented in each line, then the fold enrichment in each cluster is represented. Yellow represents a high-fold enrichment whereas purple represents a low-fold enrichment.

The GO term immune response was the most enriched term in Cluster 4 (supplemental Fig. S3 and S4, supplemental Table S4), indicating that aged OPCs acquire expression of proteins associated with immune functions in homeostasis such as CD74, B2M or TAP1. This aligns with RNA sequencing data (14, 15) and is common with other adult stem cells (48, 49). The expression of immune-associated proteins and functions in OPCs have previously being shown in adult OPCs in response to demyelination in toxin-induced models (2), experimental autoimmune encephalomyelitis (EAE) (50, 51) and in MS lesions (52). We cannot entirely exclude the possibility that this is because of immune cell contamination of our samples, but because the levels of microglia and red blood cell contamination were similar in both adult OPCs preparations (Fig. 1*B*), these data suggest an OPC subpopulation with an immune-associated profile.

##### Protein Expression Changes Between Young and Aged Adult OPCs

**Fig. 6:**
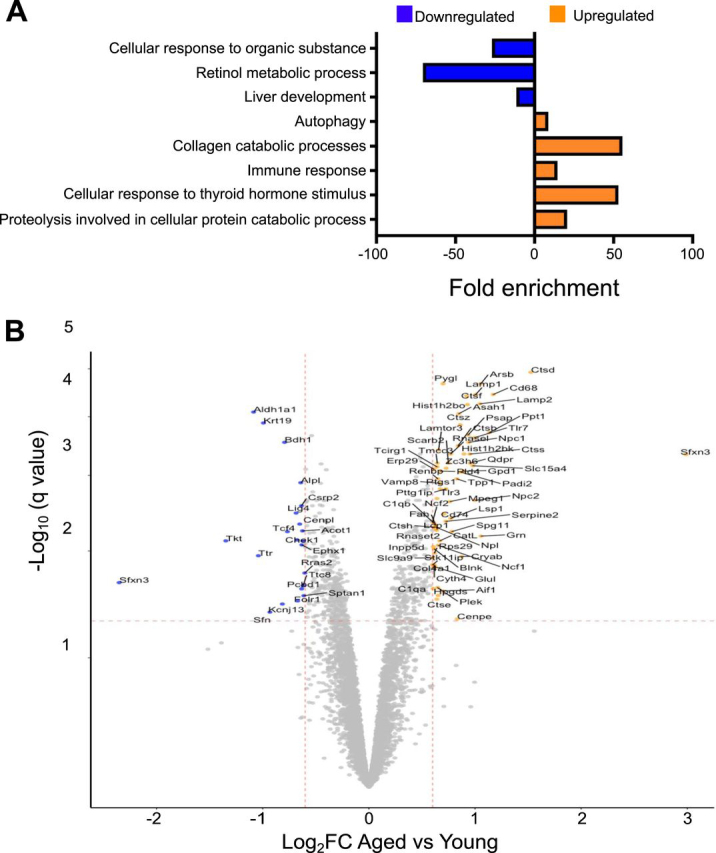
**Proteome changes between young and aged OPCs.***A*, Bar graph showing the GO Biological processes that are enriched among those proteins that have an FDR < 0.05 and more than 1.5 FC between young and aged OPCs. Orange represents the GO terms enriched among the proteins up-regulated (orange) and downregulated (blue) in aged OPCs. *B*, Volcano plot illustrating differential expression of proteins between young and aged OPCs. Colored proteins are significantly regulated with FDR < 0.05. Blue in indicates a Log_2_ change < (− 0.6) whereas orange refers to proteins with Log_2_ change >0.6. The names of the significantly regulated proteins are included in the volcano plot.

Remyelination is a highly efficient process in young adults, but its efficiency declines with ageing (5, 10, 15). To explore differences that might contribute to the aged-related decline in remyelination we compared the log_2_ intensity of the 6842 proteins present in at least 5 biological replicates detected in young and aged OPCs. We detected 659 proteins with a significant expression change (FDR 5%): 319 proteins were increased, whereas 340 were decreased in aged OPCs compared with young adult OPCs (supplemental Table S5). GO analysis revealed that increased proteins are involved in positive regulation of TOR signaling, actin polymerization or depolymerization, catabolic processes and autophagy, whereas decreased proteins are involved in retinol metabolism, responses to metal ions and mitotic nuclear division (supplemental Fig. S7). Of the 659 proteins, only 89 had a greater than 1.5-fold change in expression (supplemental Table S4). Of those 89 proteins, 67 were increased and were related to autophagy, immune responses and thyroid hormone signaling, whereas 22 were decreased and were involved with responses to organic substances or retinol metabolism (Fig. 6*A*).

Among the proteins decreased in aged OPCs are proteins involved in actin stabilization (*e.g.* SPTAN1); ALDH1A1, the enzyme responsible for the synthesis of 9-cis-retinoic acid and which promotes OPC differentiation during CNS remyelination (38); enzymes involved in matrix mineralization (*e.g.* ALPL (alkaline phosphatase)) (53); FOLR1, mutations in which have been linked to myelination deficits (54); and TCF4, which is involved in stage-specific regulation of OPC differentiation (55). Conversely, lysosomal enzymes (*e.g.* LAMTOR3, LAMP1, LAMP2, NPC1, NPC2) and cathepsins (*e.g.* CTSD, CTSB, CTSE, CTSH, CTSS, CTSZ), together with enzymes involved in the sphingolipid synthesis and modification of proteins such as citrullination (*e.g.* ARSB, PSAP, PPT1, ASAH1 AND PADI2) are increased in aged OPCs (Fig. 6*B*, supplemental Table S5).

##### Changes in the Myelin Sheath Components Within Aged OPCs

**Fig. 7:**
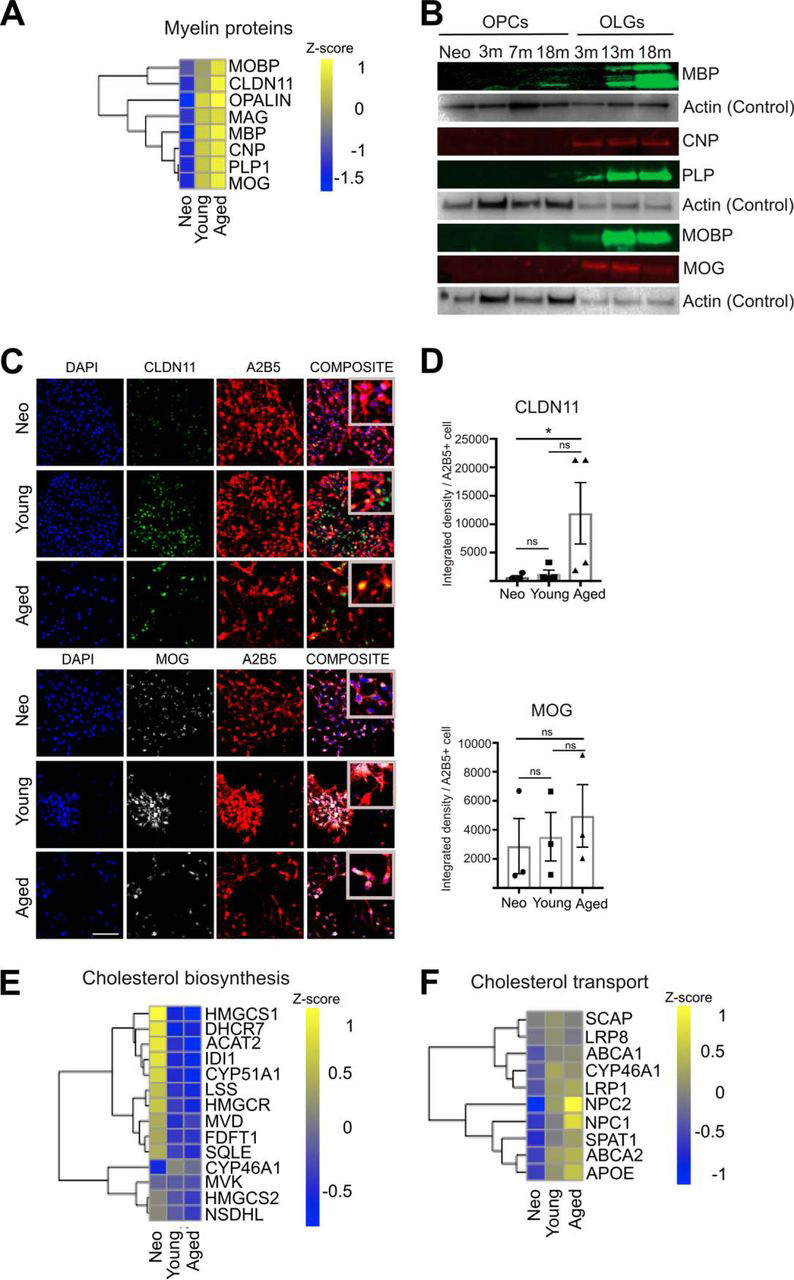
**Changes in myelin proteins and cholesterol metabolism related proteins in aged OPCs.***A*, Heat map showing the increased expression of myelin proteins in aged OPCs. Z score indicates the mean of six independent biological replicates of the proteomics dataset. Lower expression is represented in blue, and higher expression is shown in yellow. *B*, Representative Western blotting showing myelin protein expression in A2B5^+^ OPCs and MOG^+^ oligodendrocytes from different ages. *C*, Representative immunocytochemistry images of purified OPCs cultured for 6 days *in vitro* and immunostained for CLDN11 and MOG. *D*, Bar graphs indicating IHC quantification of myelin protein intensity per A2B5^+^ cells across the different age groups (each biological replicate is represented by a single dot) Scale bar 100 μm (*n* = 3, Mean ± S.E.M. shown). *E*, *F*, Heat maps showing the expression changes for proteins involved in cholesterol biosynthesis or cholesterol transport, respectively. Z score indicates the mean of six independent biological replicates and lower expression is shown in blue, and higher expression is shown in yellow.

Aged OPCs exhibited not only a decrease in proteins involved in stem cell maintenance (Fig. 5*B*) but also an increase in myelin proteins characteristic of differentiated oligodendrocytes (Fig. 7*A*–7*D* and supplemental Fig. S8). PLP, MOG, MOBP, MBP, and CNP showed a trend toward an increase in expression in aged OPCs by Western blotting but this trend was not significant, probably because of a lower sensitivity of the Western blotting to detect the subtler changes in the OPCs. On immunohistochemistry, CLDN11 (Fig. 7*C*, 7*D*) (Kruskal-Wallis *p* = 0.0274*, Dunn's Multiple comparison test neonatal *versus* young *p* = 0,9999, neonatal *versus* aged *p* = 0.0412*, young *versus* aged *p* = 0.2813) was significantly increased with ageing, whereas MOBP (Fig. 3*D*, 3*E*), MOG (1-way ANOVA *p* = 0.3087) (Fig. 7*C*, 7*D*), MBP (Kruskal Wallis *p* = 0.1097) and CNP (1-way ANOVA *p* = 0.5790) (supplemental Fig. S8B) were not significant. This increase in myelin protein expression in OPCs with ageing is unlikely to be caused by oligodendrocyte contamination during the isolation process because both preparations of adult OPCs contained only 2% A2B5^+^MOG^+^ cells and 0.4% A2B5^−^MOG^+^ cells (Fig. 1*B*). Moreover, the expression of PLP (1-way ANOVA *p* = 0.3557), MOG (1-way ANOVA *p* = 0.0012**), MOBP (1-way ANOVA *p* = 0.0458*), CNP (Kruskal-Wallis *p* = 0.0187*) and MBP (Kruskal-Wallis *p* = 0.0231*) in aged OPCs was at considerably lower levels than those associated with mature oligodendrocytes (Figs. 7*B*, supplemental Fig. S8A) (56, 57).

Important changes in the expression of enzymes involved in cholesterol biosynthesis also occurred with OPC ageing. Cholesterol synthesis enzymes (*e.g.* HMGS1, HMGS2, HMGR, DHCR7, DHCR24) and the rate-limiting enzyme for the synthesis of cholesterol, FDFT1 ((58) are significantly decreased in adult OPCs compared with neonatal OPCs (Fig. 7*E*). In contrast, proteins involved in the intracellular transport of cholesterol (*e.g.* NPC1 and NPC2), as well as APOE, the main protein responsible for cholesterol transport in the CNS, were higher in aged OPCs (Fig. 7*F*). This may be related to the completion of developmental myelination, because following this process cholesterol synthesis is decreased by around 85% (59).

##### High-Throughput Proteomics Suggests Links Between Aged OPCs and Neurodegenerative Diseases

Ageing is the highest risk factor for the development of several neurodegenerative diseases such as Alzheimer's disease (AD), Parkinson's disease (PD) and Huntington's disease (HD). Most of these diseases are characterized by the misfolding and deposition of proteins such as amyloid-β, tau, α-synuclein, TDP-43 and huntingtin. Although most research has focused on protein accumulation in neurons, several recent publications suggest that other cell types such as microglia, astrocytes, pericytes and oligodendrocytes are also involved in the development of neurodegenerative diseases (60, 61, 62, 63, 64, 65, 66).

**Fig. 8:**
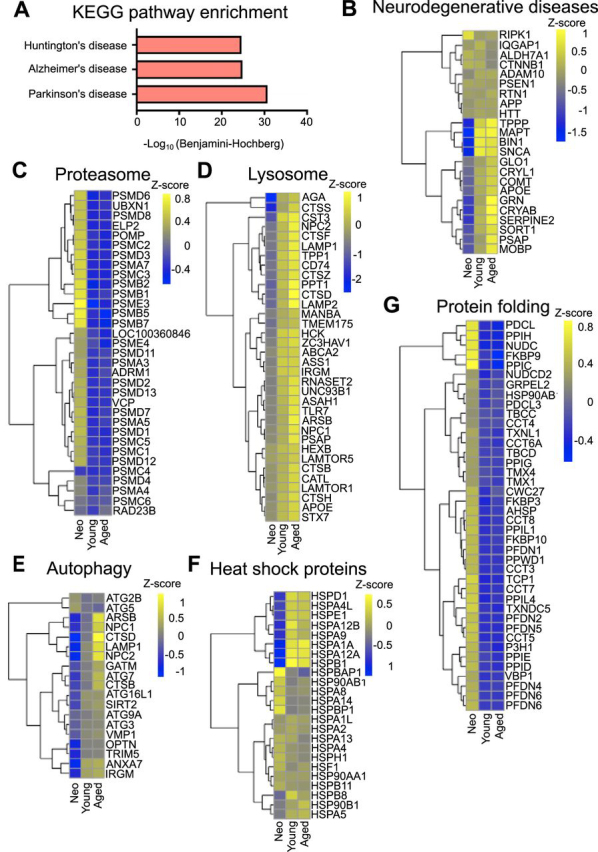
**Loss of proteostasis in aged OPCs and links to other neurodegenerative diseases.***A*, Bar graph showing the KEGG pathway enrichment of terms associated with neurodegenerative diseases within cluster 1. *B–G*, Heat maps showing the expression changes for proteins in OPCs for proteins involved in (*B*) different neurodegenerative diseases, *C*, proteasome, *D*, lysosome, *E*, autophagy, *F*, heat shock and *G*, protein folding related proteins, respectively. Z score indicates the mean of six independent samples per age group; lower expression is represented in blue, and higher expression is shown in yellow.

A striking feature in the KEGG pathways analysis of our data was the enrichment of proteins associated with AD, PD and HD in Cluster 1, the cluster that includes proteins that are increased in young and aged OPCs compared with neonatal OPCs (Fig. 8*A*, supplemental Fig. S4). Adult (young and aged) OPCs show higher expression than neonatal OPCs of some of the proteins associated with AD, PD, HD, and MS (*e.g.* TAU (67, 68), APOE (69), CRYAB (70, 71, 72, 73), TPPP (74), and SNCA (75, 76) (Fig. 8*B*).

**Fig. 9:**
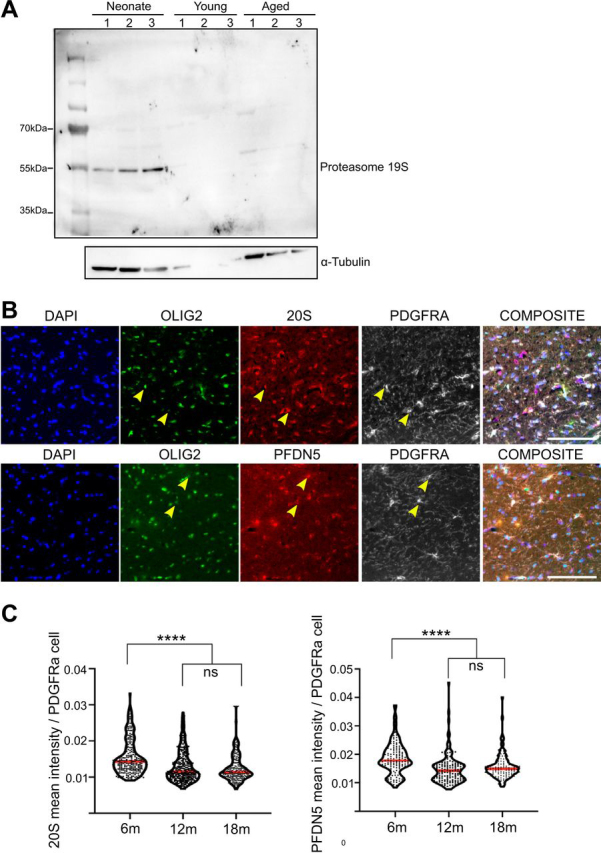
**Proteasome 19S and 20S and protein folding protein PFDN5 expression is decreased in aged OPCs.***A*, Western blotting showing the expression of proteasome 19S in neonatal, young and aged OPCs. Three replicates per age are loaded in each case (*n* = 3). *B*, Immunohistochemistry of 6-month-old brains showing the expression of proteasome 20S and PFDN5 (red) in OPCs identified by the expression of Olig2 (green) and PDGFRα cells (gray). Yellow arrows indicate examples of OPCs expressing either 20S or PFDN5. Scale bar 100 μm. *C*, Violin plots showing the quantification of the mean intensity for 20S and PFDN5 per PDGFRα^+^ cell. Each dot represents 1 cell and the red line represents the median intensity across the different cells in each age (*n* = 2 animals per age; for 20S *n* = 233 (6 m), *n* = 287 (12 m) and *n* = 264 (18 m) and for PFDN5 *n* = 132 (6 m), *n* = 143 (12 m) and *n* = 115 (18 m) cells have been quantified per age).

Dysregulation of the normal mechanisms by which aggregates of misfolded proteins are cleared involving the proteasome, autophagy and chaperone-mediated refolding/degradation is associated with neurodegeneration (47). We found that aged OPCs also have an altered protein homeostasis system. For example, heat shock proteins HSPD1 and HSPA9, as well as autophagy and lysosome-related proteins are increased with ageing (Fig. 8*D*, 8*E*, 8*F*), in contrast to the previously described age-associated decline in autophagy and lysosomes (77). On the other hand, the proteasome proteins and proteins involved in the control of protein folding are significantly decreased in aged OPCs (Fig. 8*C*, 8*G*). The decreased expression of proteasome and protein folding-associated proteins was further validated by Western blotting and immunohistochemistry. Western blotting data showed that the expression of proteasome 19S was decreased in young and aged OPCs when compared with neonatal OPCs (Fig. 9*A*). Moreover, expression of the proteasome 20S as well as prefoldin subunit 5 (PFDN5), a molecular chaperone that binds and stabilizes newly synthesize proteins allowing them to fold correctly, was also examined by immunohistochemistry (Fig. 9*B*). Both, proteasome 20S (Kruskal-Wallis *p* = <0.0001****, Dunn's Multiple Comparison test 6m *versus* 12m *p* < 0.0001****, 6m *versus* 12m *p* < 0.0001****, 12m *versus* 18m *p* = 0.9999) and PFDN5 (Kruskal-Wallis *p* = <0.0001****, Dunn's Multiple Comparison test 6m *versus* 12m *p* < 0.0001****, 6m *versus* 12m *p* < 0.0001****, 12m *versus* 18m *p* = 0.1069) decreased expression (measured as mean intensity of the protein per PDGFRα+ cell) in OPCs at 12 or 18 months of age when compared with OPCs at 6 months of age. However, no differences were detected in proteasome 20S or PFDN5 between 12 and 18 months old OPCs (Fig. 9*C*). These data suggest that aged OPCs may also be vulnerable to the deleterious consequences of aggregation-prone proteins and therefore have a potential role in other neurodegenerative diseases.

## DISCUSSION

Recent efforts to understand the molecular changes occurring in the ageing brain have often used large-scale transcriptome analysis (2, 12, 13, 14, 42, 78, 79). In contrast, there are relatively few proteome data sets relating to brain cell type specific expression patterns (17), myelin (80), OPC differentiation to oligodendrocytes (19), ES differentiation into OPCs (18) or developmental changes in neurons (81). Although transcriptomic studies provide valuable insights, most transcripts require translation to protein to deliver biological function of the expressed gene. In addition, translation of a gene can be altered at different stages, including initiation, elongation or termination by a range of mechanisms. Translational regulatory mechanisms can affect mRNAs globally or can affect a subset of mRNAs or a specific mRNA influencing mRNA to protein transition. For example, mRNAs can be sequestered to in stress granules or P bodies limiting their accessibility, translation initiation factors such as eIF2α can be subjected to post-translational modifications affecting the ability of Met-tRNAi binding to ribosomes, trans-binding proteins can bind to mRNA inhibiting translation initiation and microRNAs can lead to a series of RNA degradative mechanisms (82). The different translational regulatory mechanism can thwart mRNA translation, leading to a poor correlation between transcriptome and proteome. As such, protein expression studies are critical to define the functional state of cell populations. To better understand the age-linked protein changes in OPCs that may contribute to remyelination failure in MS, we performed a proteome analysis of OPCs from neonatal, young adult and aged rats using a relative quantitative approach that allowed comparison of OPC protein expression levels across the three age groups. We used *ex vivo* freshly isolated OPCs to avoid *in vitro* artifacts and culture-based selection of “fittest” cells based on the environment provided. Although relatively pure OPC populations from the neonatal CNS can be isolated and grown in tissue culture, the same is not true of adult and especially aged OPCs, the culturing of which is notoriously challenging. Despite the high purity obtained in neonatal OPCs (90% A2B5^+^ cells), the same isolation methodology used for young and aged tissue renders a population significantly enriched in OPCs but with a higher microglia and red blood cell contamination. Therefore, despite the very stringent “cut-off” for downstream analysis, we caution that some of the altered protein expression may be because of other cell type contaminants rather than intrinsic changes in OPCs *per se*.

One of the most striking features of the ageing OPC proteome is that neonatal OPCs differ in ∼50% of the proteome from their young and aged counterparts. This difference raises an important point for experiments related with the therapeutic targeting of OPC differentiation as a treatment for MS. Despite MS being largely a disease of adulthood and ageing, most *in vitro* differentiation assays have focused on developmental OPCs (40, 83, 84, 85). Therefore, when screening for pro-differentiating agents it is necessary to validate findings obtained with neonatal OPCs using (aged) adult OPCs and *in vivo*, especially taking to account that MS is a disease of adulthood. The proteomes of young and aged OPCs were highly correlated, with 659 proteins significantly altered and only 89 proteins showing more than a 1.5-fold change. Despite the large number of proteins detected, the ageing OPC proteome is not complete in our samples: low abundance proteins such as transcription factors are underrepresented and the analysis of the different isoforms as well as the potential changes in post-translational modifications such as phosphorylation, citrullination or ubiquitination have not been assessed.

In common with other adult stem cells (56, 57), adult OPCs not only decrease the expression of proteins involved in stem cell maintenance, but also acquire a higher expression of proteins known to be markers of the terminal state of their lineage, the mature oligodendrocytes. These data suggest that with ageing, OPCs undergo a degree of differentiation ‘drift’, despite their ability to give rise to new oligodendrocytes being impaired with ageing (9, 10). We also observed that unlike myelin proteins, cholesterol biosynthesis related enzymes were decreased after development and in the aged OPCs, as described for aged astrocytes (86). In addition, aged phagocytes have a reduced capacity to clear and recycle cholesterol back to the extracellular space (8), which may contribute to the diminished cholesterol availability in the aged brain. This reduced cholesterol availability may influence the capacity of aged OPCs to differentiate into myelinating oligodendrocytes and contribute to age-related remyelination failure. Cholesterol supplementation in the diet increases the thickness of myelin during remyelination in mice that were 8–10 weeks of age suggesting that OPCs behavior is influenced by dietary cholesterol (87). This reduction in cholesterol together with the enhanced expression of myelin proteins may also be key not only for age-associated remyelination failure but also for new myelinating oligodendrocyte formation during myelin plasticity (88, 89, 90).

A feature of ageing populations is the rise in the number of people with neurodegenerative diseases such as AD and PD. OPCs play a key role in primary conditions of myelination, but their role in classical neurodegenerative diseases is little explored. Our proteome analysis showed that aged OPCs increased the expression of proteins involved in AD, PD, or HD and have an altered protein homeostasis system suggesting a potential accumulation of protein aggregates like those prevalent in ageing neurons. AD patients and AD mouse models show white mater alterations, fewer Olig2^+^ cells (64, 91, 92, 93) and an increased number of senescent OPCs associated with amyloid plaques (94). Moreover, aged OPCs have a higher expression of proteins like CRYAB and QDPR, like that which occurs in OPCs and oligodendrocytes from AD patients (73). Multiple system atrophy (MSA), is characterized by abnormal accumulation of α-synuclein in oligodendrocytes that causes myelin and neuron degeneration (65, 95). The overexpression of α-synuclein in stem cell-derived oligodendrocytes impairs myelin formation (96) and its aggregates interfere with the expression of myelination associated mRNAs *in vitro* (97). Our data suggests that the α-synuclein aggregates in OPCs (97, 98) increase with ageing, potentially contributing to OPC differentiation impairment. Moreover, the aggregation of misfolded proteins may play a key role in altering the basal energy state of the cell and therefore contribute to age-associated remyelination failure. Misfolded protein aggregation might arise in aged OPCs because of the increased expression of PADI2, an enzyme responsible for the citrullination of different proteins in the brain that has also been recently implicated in OPC differentiation (99). The changes in positive charges of proteins like MBP, because of the conversion of arginine into citrulline, changes their structure leading to increased demyelination (100), pronounced denaturation and protein misfolding (101). Therefore, citrullination is considered a hallmark of different neurodegenerative diseases (102, 103, 104). Moreover, MBP citrullination has been recently shown to trigger autoimmune demyelination in a model of MS (105) and its increased expression in aged OPCs may therefore contribute to protein aggregation and remyelination failure. In response to protein aggregation, an increase in lysosomal and autophagy associated proteins was observed in aged OPCs contrary to what has been described in ageing (77). Lysosomes are also considered primary sensors of the cell state, as they are able to sense energy metabolic state and are a component of primary nutrient sensing pathway. Therefore, the increase in lysosomal proteins in aged OPCs could be associated with increased OPC quiescence. As lysosomes are key in maintaining neural stem cell quiescence (106), a dysregulation and accumulation of storage material in the cell that requires increase lysosome biogenesis (107, 108) as occurs in neuronal lipofuscinosis, could signal OPC senescence. Thus, this change reflect an adaptation of the lysosome to changes in the energy state of aged OPCs or to a higher inflammatory environment, similar to that shown in activated phagocytes, cancer cells and other inflammatory diseases (109, 110, 111). These increases in lysosomes and autophagy could also be a cell-intrinsic compensatory mechanism in response to ageing cues (77). However, changes in lysosomal proteins do not necessarily always correlate with lysosomal function as shown for neurons in models of AD (112).

In summary, the analysis of the proteome of OPCs in the context of ageing provides a unique tool to understand the protein changes that underlie age-associated remyelination failure, and potentially other physiological and pathophysiological roles of these abundant and widely distributed CNS cells. We observed key changes in proteins involved in metabolism, immune response and actin cytoskeleton that may influence OPC remyelination biology. In addition, the study of the OPC proteome in the context of ageing provides new indications that OPCs, and the alterations they undergo with ageing, may be relevant for the development of other neurodegenerative diseases.

## DATA AVAILABILITY

The mass spectrometry proteomics data have been deposited to the ProteomeXchange. Consortium via the PRIDE partner repository (113) with the dataset identifier PXD013708.
