# Supplementary material for: Changes in the Oligodendrocyte Progenitor Cell Proteome with Ageing
Source: Mol Cell Proteomics. 2020 Nov 23;19(8):1281–302. doi: 10.1074/mcp.RA120.002102 (PMC8015006; doi:10.1074/mcp.RA120.002102)
Supplement: Supplementary file 1 [file mmc1.zip › 160324_0_supp_515398_q95d33.pdf]

A

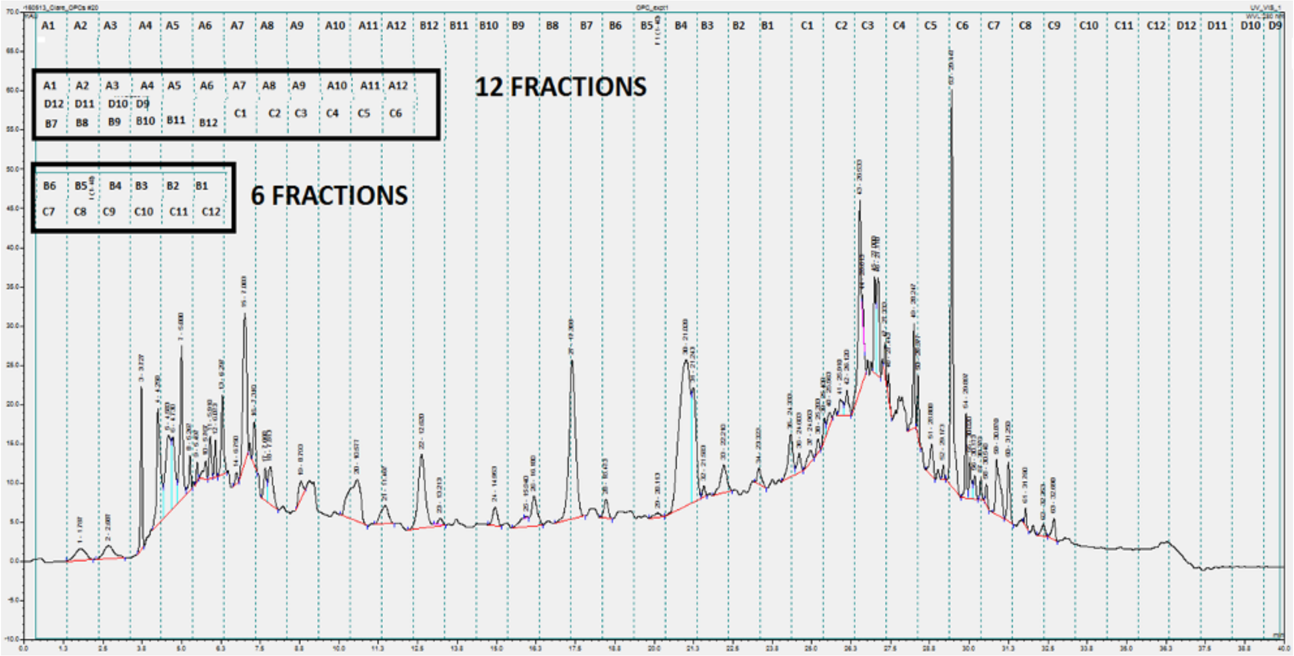

A1 A2 A3 A4 A5 A6 A7 A8 A9 A10 A11 A12 B12 B11 B10 B9 B8 B7 B6 B5 B4 B3 B2 B1 C1 C2 C3 C4 C5 C6 C7 C8 C9 C10 C11 C12 D12 D11 D10 D9
A1 A2 A3 A4 A5 A6 A7 A8 A9 A10 A11 A12
D12 D11 D10 D9
B7 B8 B9 B10 B11 B12 C1 C2 C3 C4 C5 C6
12 FRACTIONS
B6 B5 B4 B3 B2 B1
C7 C8 C9 C10 C11 C12
6 FRACTIONS

B

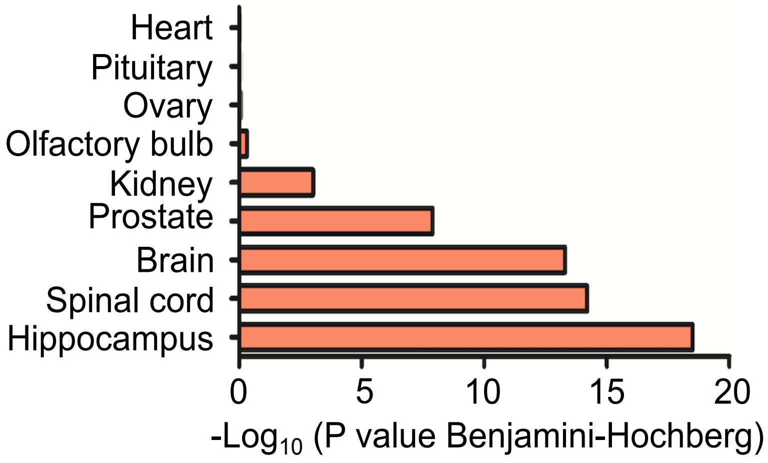

Heart
Pituitary
Ovary
Olfactory bulb
Kidney
Prostate
Brain
Spinal cord
Hippocampus
0
5
10
15
20
-Log10 (P value Benjamini-Hochberg)

Fig. S1

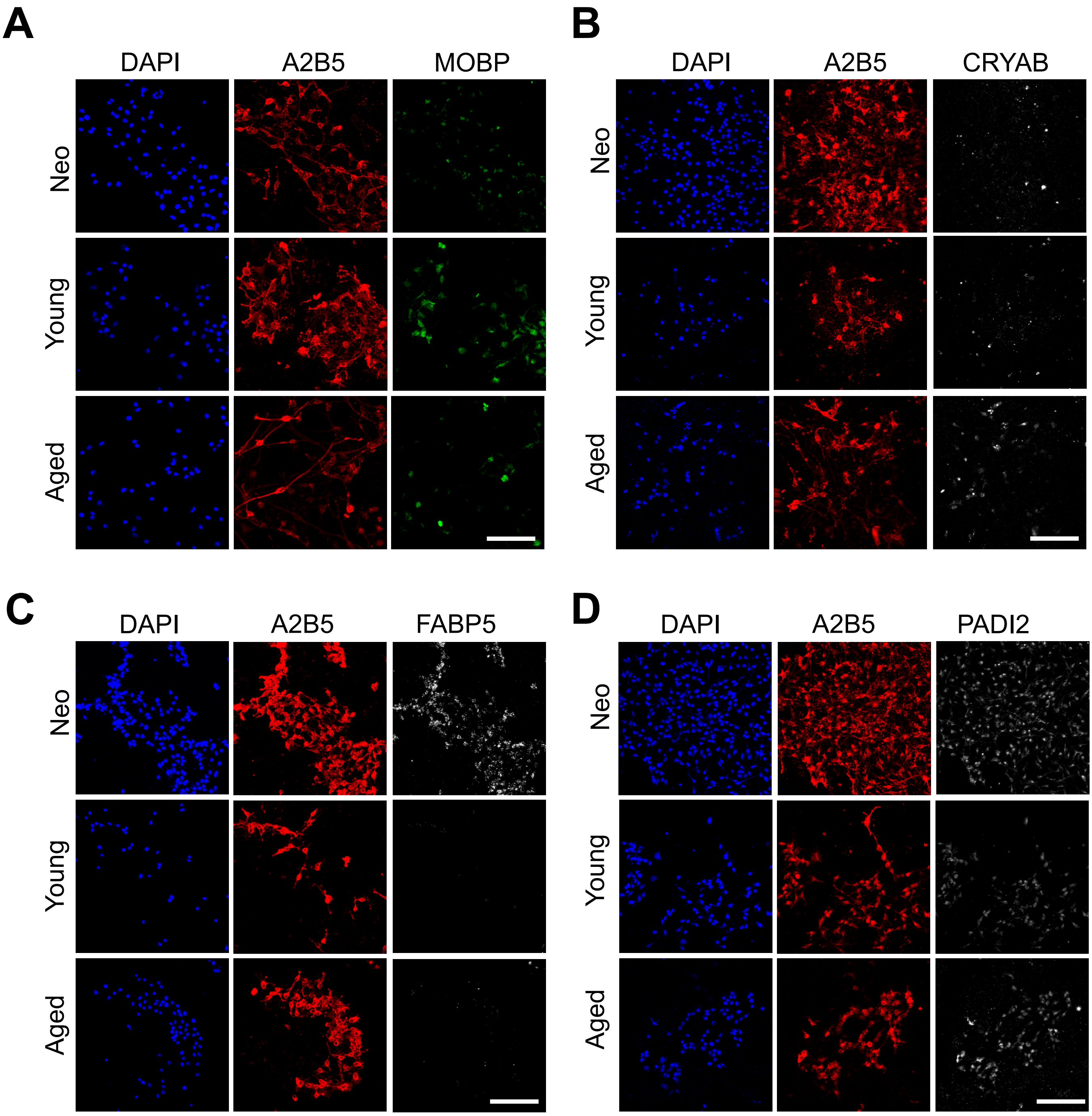
A
DAPI
A2B5
MOBP
Neo
Young
Aged
B
DAPI
A2B5
CRYAB
Neo
Young
Aged
C
DAPI
A2B5
FABP5
Neo
Young
Aged
D
DAPI
A2B5
PADI2
Neo
Young
Aged

Fig. S2

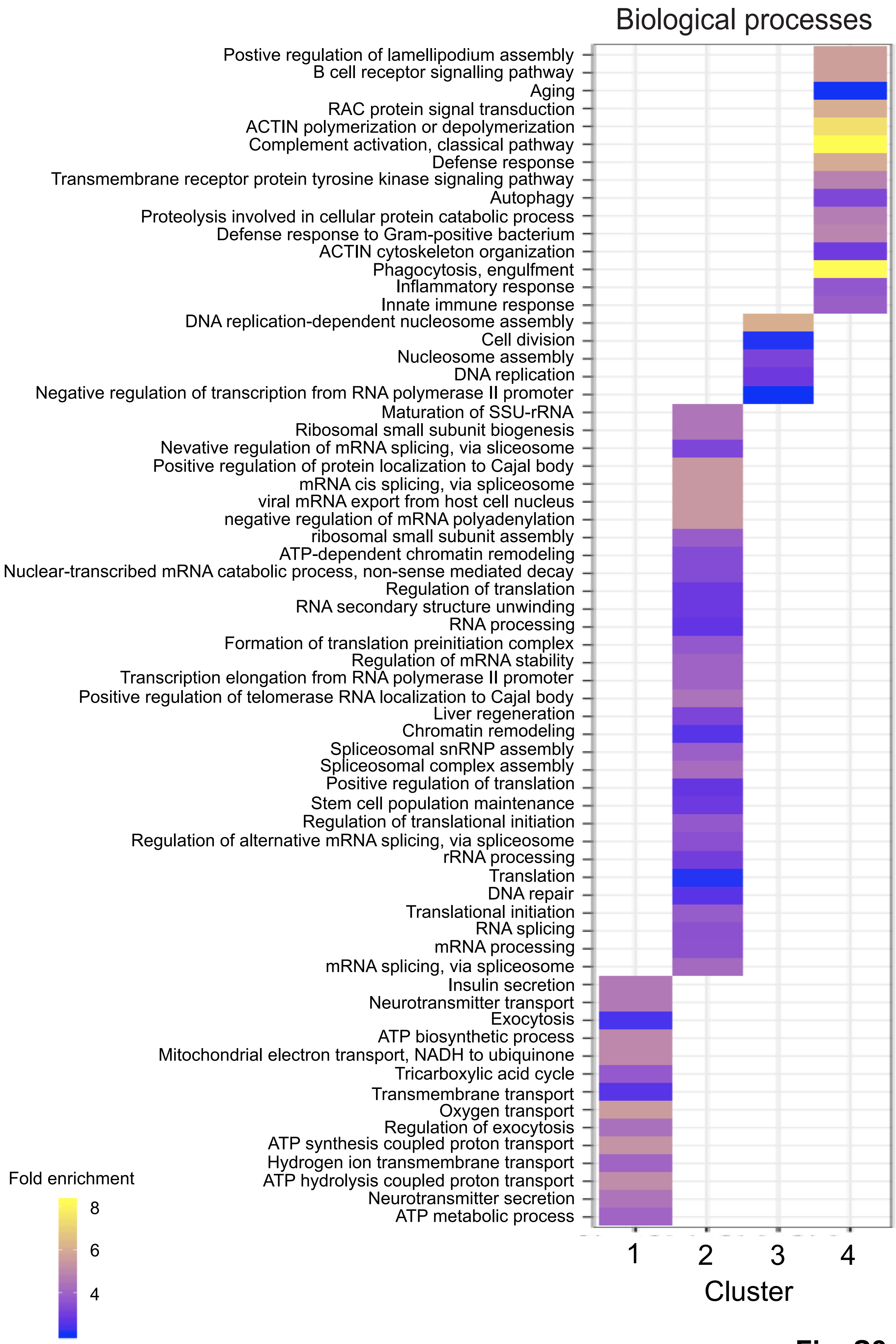
Biological processes
Postive regulation of lamellipodium assembly
B cell receptor signalling pathway
Aging
RAC protein signal transduction
ACTIN polymerization or depolymerization
Complement activation, classical pathway
Defense response
Transmembrane receptor protein tyrosine kinase signaling pathway
Autophagy
Proteolysis involved in cellular protein catabolic process
Defense response to Gram-positive bacterium
ACTIN cytoskeleton organization
Phagocytosis, engulfment
Inflammatory response
Innate immune response
DNA replication-dependent nucleosome assembly
Cell division
Nucleosome assembly
DNA replication
Negative regulation of transcription from RNA polymerase II promoter
Maturation of SSU-rRNA
Ribosomal small subunit biogenesis
Nevative regulation of mRNA splicing, via sliceosome
Positive regulation of protein localization to Cajal body
mRNA cis splicing, via spliceosome
viral mRNA export from host cell nucleus
negative regulation of mRNA polyadenylation
ribosomal small subunit assembly
ATP-dependent chromatin remodeling
Nuclear-transcribed mRNA catabolic process, non-sense mediated decay
Regulation of translation
RNA secondary structure unwinding
RNA processing
Formation of translation preinitiation complex
Regulation of mRNA stability
Transcription elongation from RNA polymerase II promoter
Positive regulation of telomerase RNA localization to Cajal body
Liver regeneration
Chromatin remodeling
Spliceosomal snRNP assembly
Spliceosomal complex assembly
Positive regulation of translation
Stem cell population maintenance
Regulation of translational initiation
Regulation of alternative mRNA splicing, via spliceosome
rRNA processing
Translation
DNA repair
Translational initiation
RNA splicing
mRNA processing
mRNA splicing, via spliceosome
Insulin secretion
Neurotransmitter transport
Exocytosis
ATP biosynthetic process
Mitochondrial electron transport, NADH to ubiquinone
Tricarboxylic acid cycle
Transmembrane transport
Oxygen transport
Regulation of exocytosis
ATP synthesis coupled proton transport
Hydrogen ion transmembrane transport
ATP hydrolysis coupled proton transport
Neurotransmitter secretion
ATP metabolic process
1
2
3
4
Cluster
Fold enrichment
8
6
4

Fig. S3

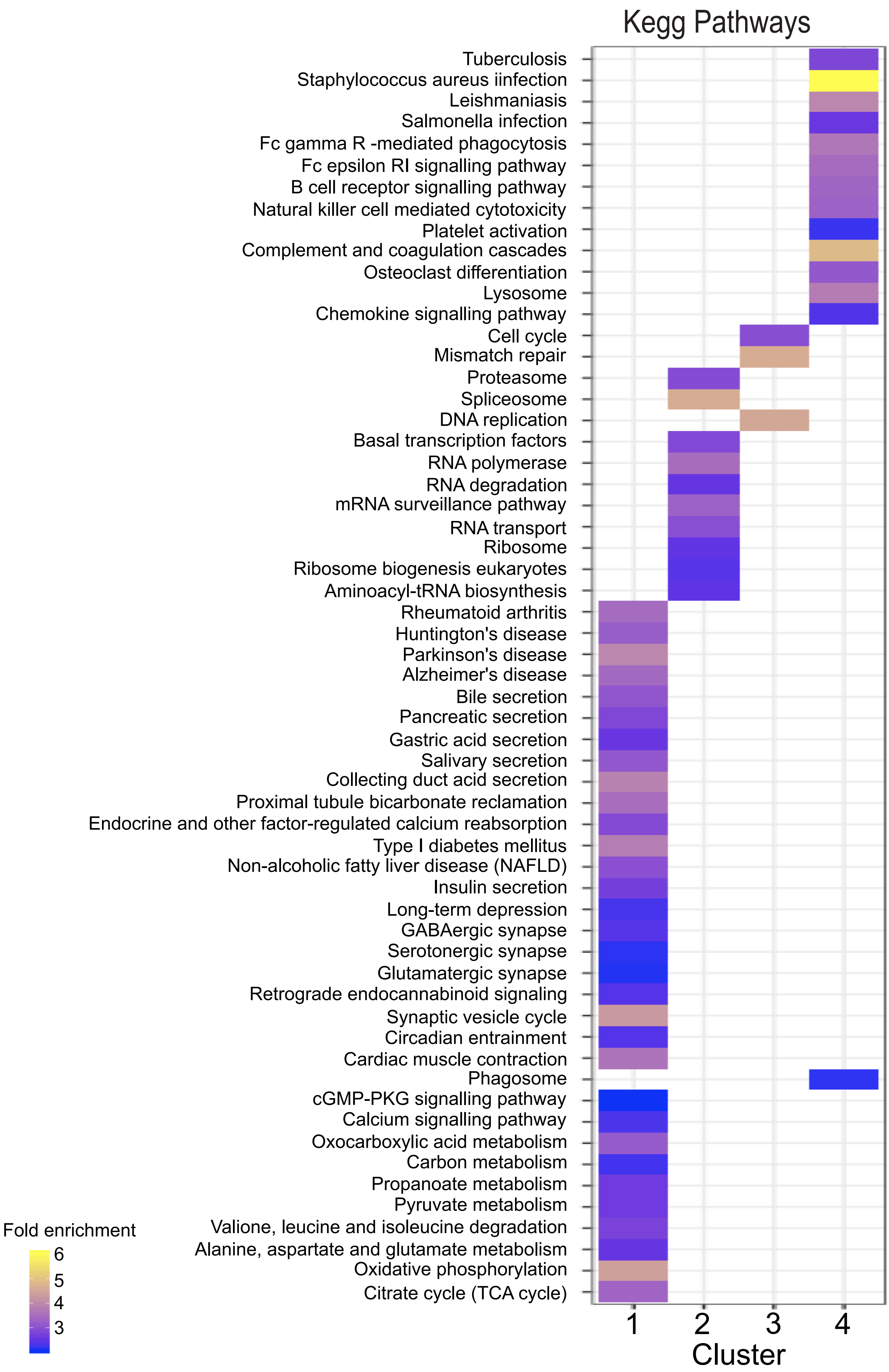
Kegg Pathways
Tuberculosis
Staphylococcus aureus iinfection
Leishmaniasis
Salmonella infection
Fc gamma R -mediated phagocytosis
Fc epsilon RI signalling pathway
B cell receptor signalling pathway
Natural killer cell mediated cytotoxicity
Platelet activation
Complement and coagulation cascades
Osteoclast differentiation
Lysosome
Chemokine signalling pathway
Cell cycle
Mismatch repair
Proteasome
Spliceosome
DNA replication
Basal transcription factors
RNA polymerase
RNA degradation
mRNA surveillance pathway
RNA transport
Ribosome
Ribosome biogenesis eukaryotes
Aminoacyl-tRNA biosynthesis
Rheumatoid arthritis
Huntington's disease
Parkinson's disease
Alzheimer's disease
Bile secretion
Pancreatic secretion
Gastric acid secretion
Salivary secretion
Collecting duct acid secretion
Proximal tubule bicarbonate reclamation
Endocrine and other factor-regulated calcium reabsorption
Type I diabetes mellitus
Non-alcoholic fatty liver disease (NAFLD)
Insulin secretion
Long-term depression
GABAergic synapse
Serotonergic synapse
Glutamatergic synapse
Retrograde endocannabinoid signaling
Synaptic vesicle cycle
Circadian entrainment
Cardiac muscle contraction
Phagosome
cGMP-PKG signalling pathway
Calcium signalling pathway
Oxocarboxylic acid metabolism
Carbon metabolism
Propanoate metabolism
Pyruvate metabolism
Valione, leucine and isoleucine degradation
Alanine, aspartate and glutamate metabolism
Oxidative phosphorylation
Citrate cycle (TCA cycle)
1
2
3
4
Cluster
Fold enrichment
6
5
4
3

Fig. S4

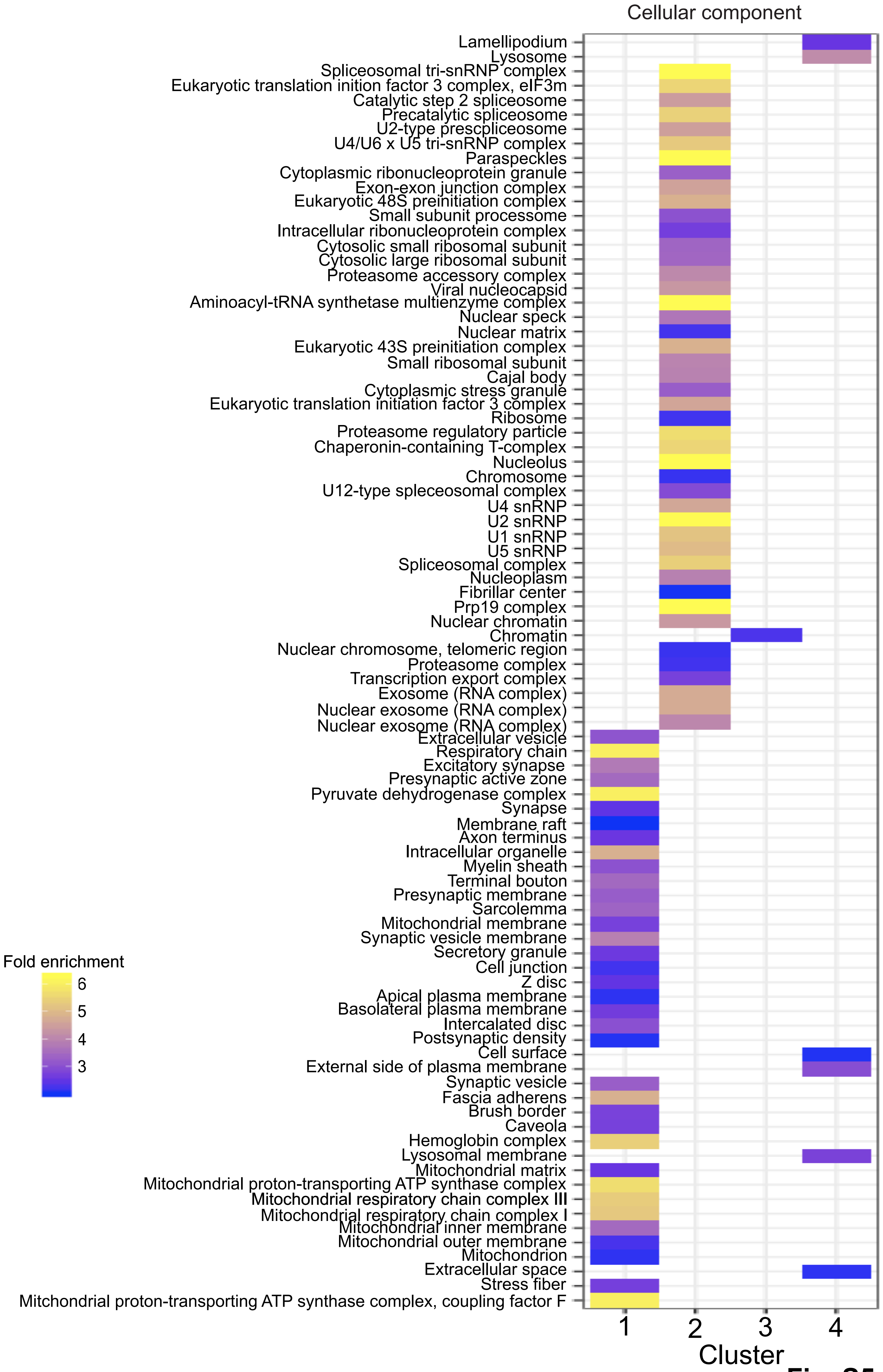
Cellular component
Lamellipodium
Lysosome
Spliceosomal tri-snRNP complex
Eukaryotic translation inition factor 3 complex, eIF3m
Catalytic step 2 spliceosome
Precatalytic spliceosome
U2-type prescpliceosome
U4/U6 x U5 tri-snRNP complex
Paraspeckles
Cytoplasmic ribonucleoprotein granule
Exon-exon junction complex
Eukaryotic 48S preinitiation complex
Small subunit processome
Intracellular ribonucleoprotein complex
Cytosolic small ribosomal subunit
Cytosolic large ribosomal subunit
Proteasome accessory complex
Viral nucleocapsid
Aminoacyl-tRNA synthetase multienzyme complex
Nuclear speck
Nuclear matrix
Eukaryotic 43S preinitiation complex
Small ribosomal subunit
Cajal body
Cytoplasmic stress granule
Eukaryotic translation initiation factor 3 complex
Ribosome
Proteasome regulatory particle
Chaperonin-containing T-complex
Nucleolus
Chromosome
U12-type spleceosomal complex
U4 snRNP
U2 snRNP
U1 snRNP
U5 snRNP
Spliceosomal complex
Nucleoplasm
Fibrillar center
Prp19 complex
Nuclear chromatin
Chromatin
Nuclear chromosome, telomeric region
Proteasome complex
Transcription export complex
Exosome (RNA complex)
Nuclear exosome (RNA complex)
Nuclear exosome (RNA complex)
Extracellular vesicle
Respiratory chain
Excitatory synapse
Presynaptic active zone
Pyruvate dehydrogenase complex
Synapse
Membrane raft
Axon terminus
Intracellular organelle
Myelin sheath
Terminal bouton
Presynaptic membrane
Sarcolemma
Mitochondrial membrane
Synaptic vesicle membrane
Secretory granule
Cell junction
Z disc
Apical plasma membrane
Basolateral plasma membrane
Intercalated disc
Postsynaptic density
Cell surface
External side of plasma membrane
Synaptic vesicle
Fascia adherens
Brush border
Caveola
Hemoglobin complex
Lysosomal membrane
Mitochondrial matrix
Mitochondrial proton-transporting ATP synthase complex
Mitochondrial respiratory chain complex III
Mitochondrial respiratory chain complex I
Mitochondrial inner membrane
Mitochondrial outer membrane
Mitochondrion
Extracellular space
Stress fiber
Mitchondrial proton-transporting ATP synthase complex, coupling factor F
1
2
3
4
Cluster
Fold enrichment
6
5
4
3

Fig. S5

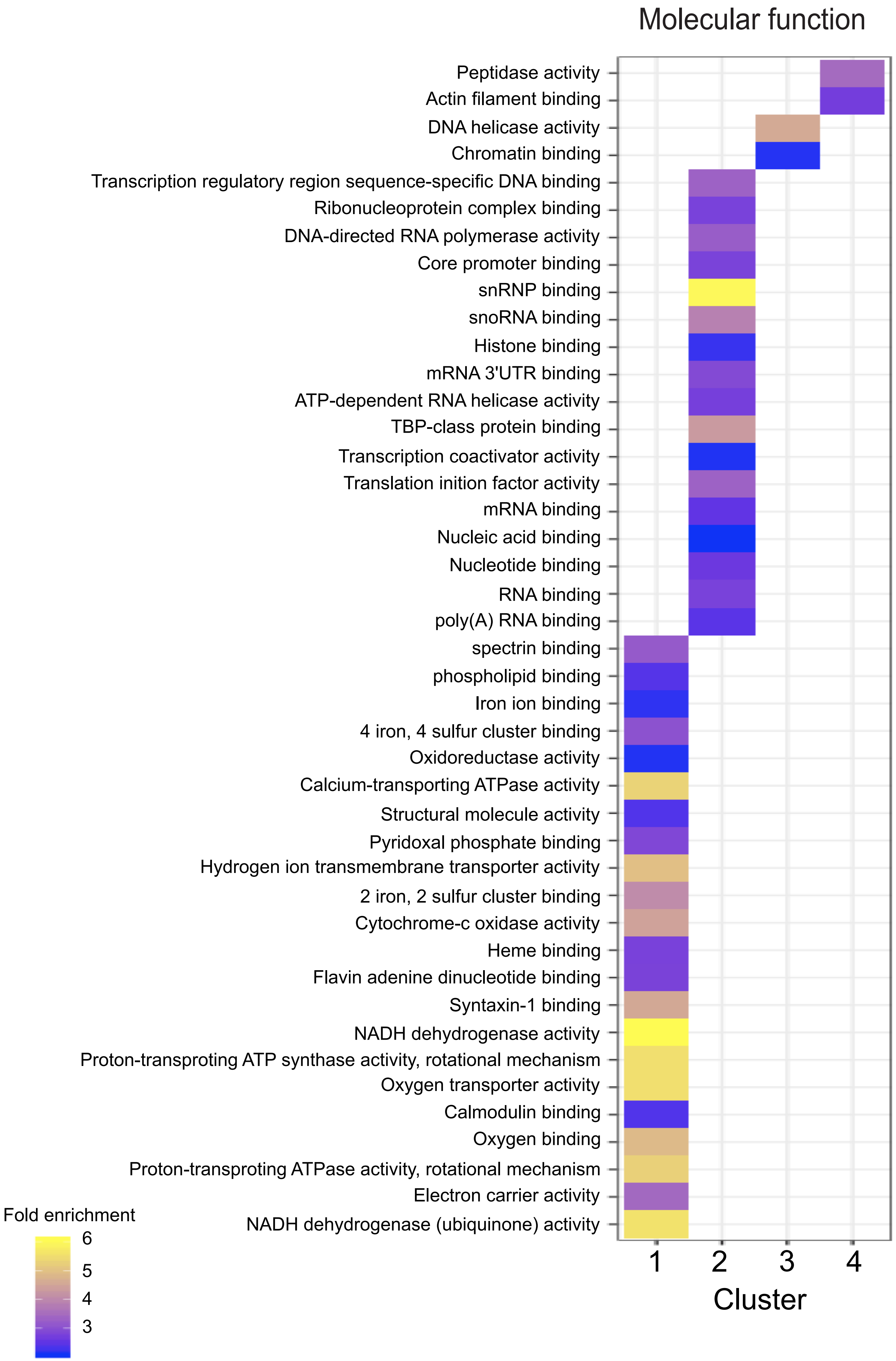

Molecular function
Peptidase activity
Actin filament binding
DNA helicase activity
Chromatin binding
Transcription regulatory region sequence-specific DNA binding
Ribonucleoprotein complex binding
DNA-directed RNA polymerase activity
Core promoter binding
snRNP binding
snoRNA binding
Histone binding
mRNA 3'UTR binding
ATP-dependent RNA helicase activity
TBP-class protein binding
Transcription coactivator activity
Translation inition factor activity
mRNA binding
Nucleic acid binding
Nucleotide binding
RNA binding
poly(A) RNA binding
spectrin binding
phospholipid binding
Iron ion binding
4 iron, 4 sulfur cluster binding
Oxidoreductase activity
Calcium-transporting ATPase activity
Structural molecule activity
Pyridoxal phosphate binding
Hydrogen ion transmembrane transporter activity
2 iron, 2 sulfur cluster binding
Cytochrome-c oxidase activity
Heme binding
Flavin adenine dinucleotide binding
Syntaxin-1 binding
NADH dehydrogenase activity
Proton-transproting ATP synthase activity, rotational mechanism
Oxygen transporter activity
Calmodulin binding
Oxygen binding
Proton-transproting ATPase activity, rotational mechanism
Electron carrier activity
NADH dehydrogenase (ubiquinone) activity
1
2
3
4
Cluster
Fold enrichment
6
5
4
3

Fig. S6

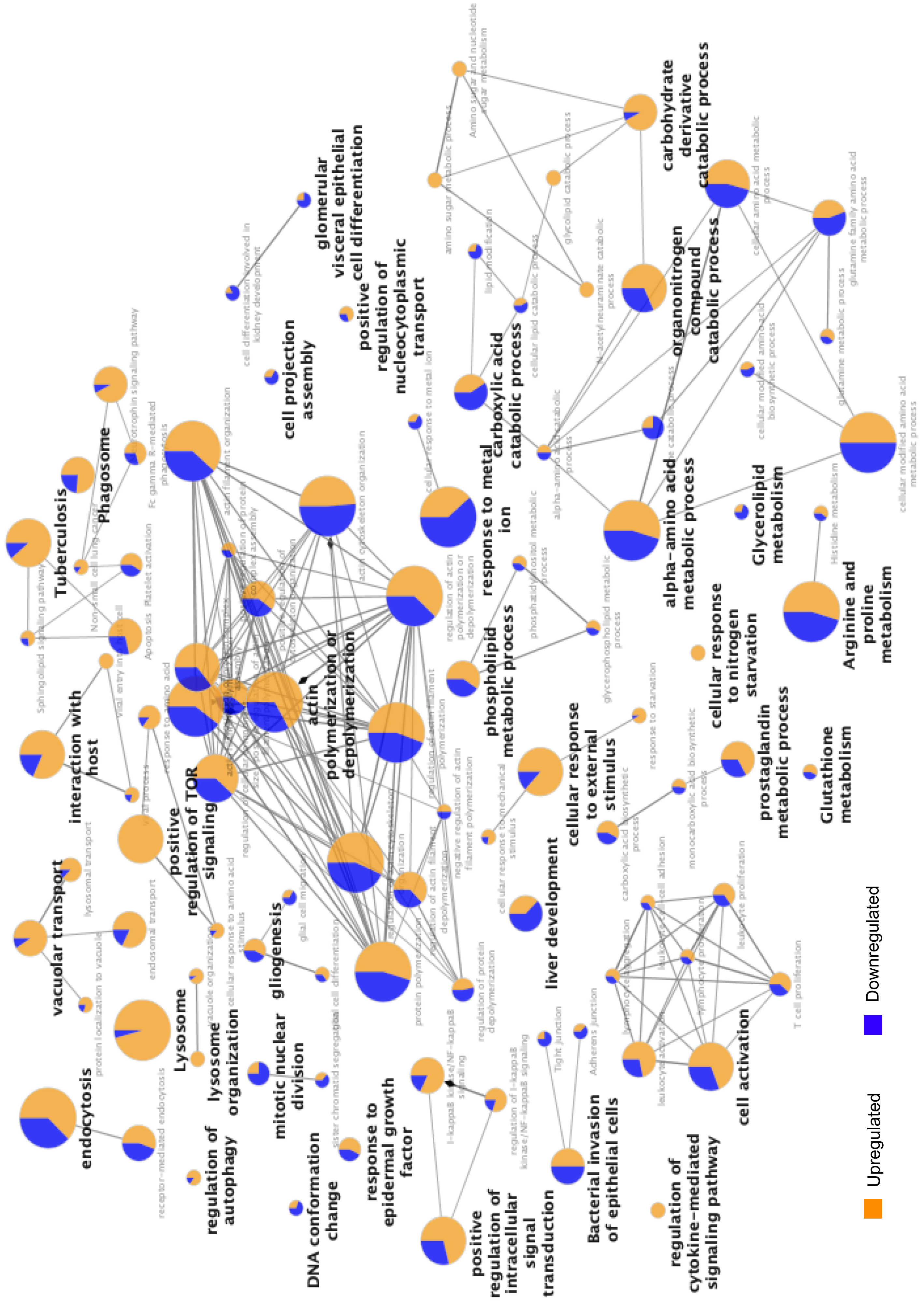
endocytosis
vacuolar transport
Lysosome
lysosome organization
regulation of autophagy
mitotic nuclear division
DNA conformation change
gliogenesis
response to epidermal growth factor
positive regulation of intracellular signal transduction
liver development
Bacterial invasion of epithelial cells
cell activation
regulation of cytokine-mediated signaling pathway
positive regulation of TOR signaling
interaction with host
Tuberculosis
Phagosome
actin polymerization or depolymerization
cell projection assembly
glomerular visceral epithelial cell differentiation
positive regulation of nucleocytoplasmic transport
response to metal ion
carboxylic acid catabolic process
phospholipid metabolic process
cellular response to external stimulus
prostaglandin metabolic process
Glutathione metabolism
cellular response to nitrogen starvation
alpha-amino acid metabolic process
organonitrogen compound catabolic process
carbohydrate derivative catabolic process
Glycerolipid metabolism
Arginine and proline metabolism
Upregulated
Downregulated

Fig. S7

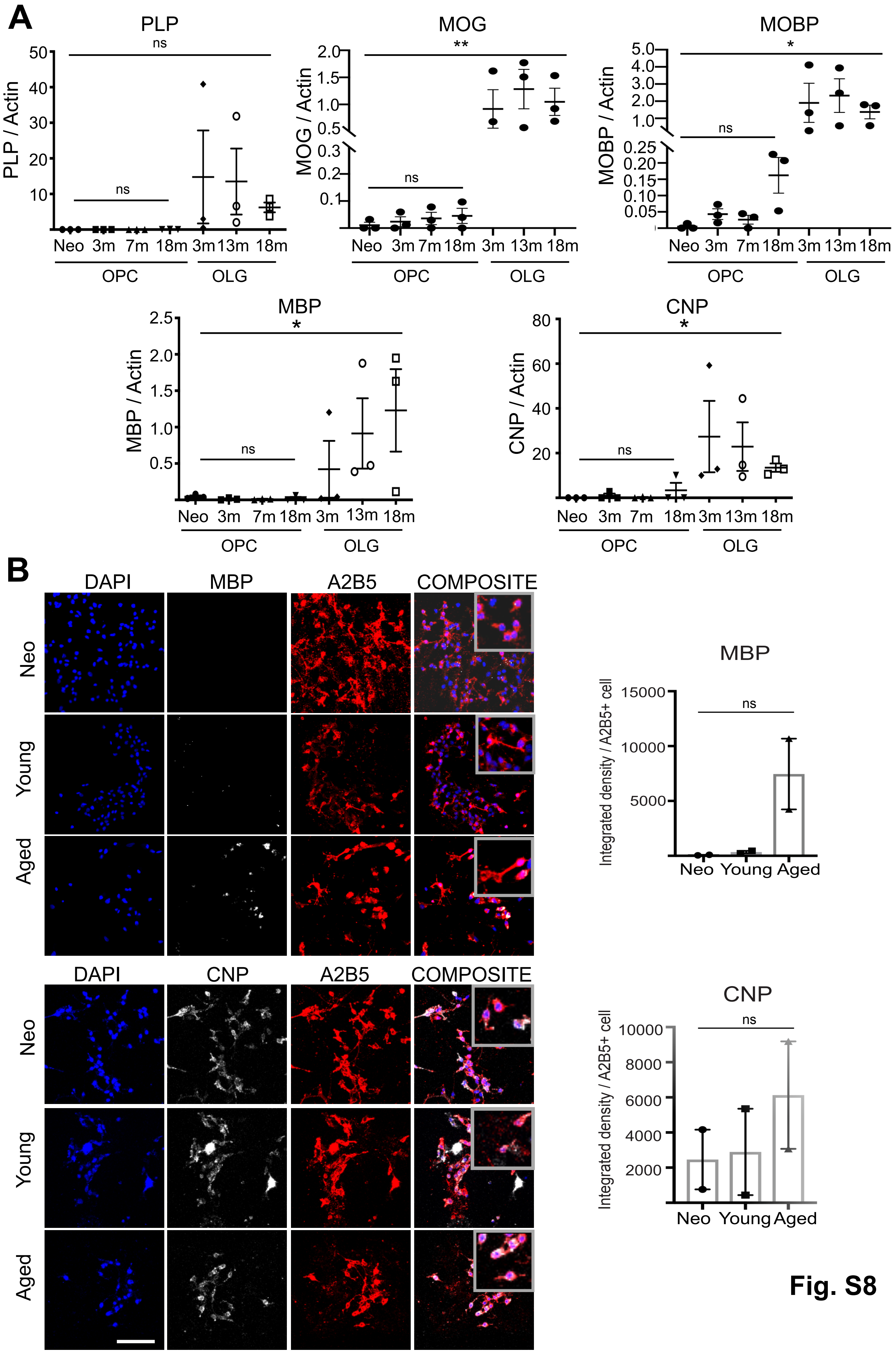
A
PLP
PLP / Actin
MOG
MOG / Actin
MOBP
MOBP / Actin
MBP
MBP / Actin
CNP
CNP / Actin
ns
**
*
Neo 3m 7m 18m 3m 13m 18m
OPC
OLG
B
DAPI
MBP
A2B5
COMPOSITE
CNP
Neo
Young
Aged
Integrated density / A2B5+ cell
Neo Young Aged

Fig. S8

SUPPLEMENTAL FIGURES AND TABLES

Figure S1: Data processing and tissue enrichment (Related to Fig. 1). (**A**) Image showing the concatenation procedure of the of the 18 fractions run through the mass spectrometer. (**B**)Bar graph showing the tissue enrichment analysis for all proteins detected using DAVID.

Figure S2: Immunocytochemistry for MOBP, CRYAB, FABP5 and PADI2 in neonatal young and aged OPCs (Related to Fig. 3). (**A**) Individual channels showing the expression of DAPI (blue), A2B5 (red) and MOBP (green) in neonatal, young and aged OPCs. (**B**) Individual channels showing the expression of DAPI (blue), A2B5 (red) and CRAYB (grey) in neonatal, young and aged OPCs. (**C**) Individual channels showing the expression of DAPI (blue), A2B5 (red) and FABP5 (grey) in neonatal, young and aged OPCs. (**D**) Individual channels showing the expression of DAPI (blue), A2B5 (red) and PADI2 (grey) in neonatal, young and aged OPCs. Scale bar 100µm.

Figure S3: GO Term Biological processes analysis of the significantly regulated proteins of the four abundance profile clusters obtained with unsupervised fuzzy C-means clustering (Related to Fig.4). Heatmaps representing the fold enrichment in the number of proteins present in each cluster that belong to the different the biological processes (in horizontal lines) using DAVID when compared to the total proteome detected. Yellow represents a higher fold enrichment and purple a lower fold enrichment. (GO terms with fold enrichment >2 and p value Benjamini-Hochberg <0.05 are indicated).

Figure S4: KEGG pathway analysis of the significantly regulated proteins of the four abundance profile clusters obtained with unsupervised fuzzy C-means clustering (Related to Fig.4). Heatmaps representing the fold enrichment in the number of proteins present in each the 4 clusters that belong to the different the KEGG pathways (in horizontal lines) using DAVID when compared to the total proteome detected. Yellow represents a higher fold enrichment and purple a lower fold enrichment. (GO terms with fold enrichment >2 and p value Benjamini-Hochberg <0.05 are indicated).

Figure S5: GO Cellular Compartment analysis of the significantly regulated proteins of the four abundance profile clusters obtained with unsupervised fuzzy C-means clustering (Related to Fig.4). Heatmaps representing the fold enrichment in the number of proteins present in each of the 4 clusters that belong to the different GO cellular components (in horizontal lines) using DAVID when compared to the total proteome detected. Yellow represents a higher fold enrichment and purple a lower fold enrichment. (GO terms with fold enrichment >2 and p value Benjamini-Hochberg <0.05 are indicated).

Figure S6: GO Molecular Function analysis of the significantly regulated proteins of the four abundance profile clusters obtained with unsupervised fuzzy C-means clustering (Related to Fig.4). Heatmaps representing the fold enrichment of the number of proteins present in each of the 4 clusters that are involved in the different molecular functions (in horizontal lines) using DAVID when compared to the total proteome detected. Yellow represents a higher fold enrichment and purple a lower fold enrichment. (GO terms with fold enrichment >2 and p value Benjamini-Hochberg <0.05 are indicated).

Figure S7. GO biological process analysis of proteins significantly regulated between young and aged OPCs (Related to Fig.6). Network analysis of the GO terms enriched in the 659 proteins with FDR < 0.05 between young and aged OPCs. The circle size is correlated to the number of proteins in each GO term. Blue and orange represent the percentage of proteins involved in that GO term that are downregulated and upregulated in aged OPCs, respectively.

Figure S8: Increased myelin protein expression with ageing (Related to Fig. 7). (**A**) Graphs showing the densitometry quantification of the expression of PLP, MOG, MOBP, MBP and CNP in OPCs at neonatal, 3m, 7m and 18m as well as oligodendrocytes at 3m, 13m and 18m (n=3, mean ± s.e.m. shown). (**B**) Immunocytochemistry and quantification of the expression of MBP and CNP per A2B5+ cells. Images show the individual channels for DAPI (blue), A2B5 (red) and MBP or CNP (grey) as well as the composite image. Scale bar 100µm. (n=2-3, mean ± s.e.m. shown).

Table S1: OPC proteome (Related to Fig.1).

Table includes the $\log_2$ change of the 7481 protein groups found across the different ages and biological replicates.

Table S2: Statistical analysis table (Related to Fig. 4).

Table includes the protein groups present in at least 5 out 6 biological replicates. It also includes the $\log_2$ change and the q value for all the statistical comparisons between young and aged OPCs with neonatal OPCs.

Table S3: Unsupervised fuzzy C-means clustering table (Related to Fig. 4).

Table includes the proteins of the different clusters, their membership to each cluster and their maximum membership together with the statistical analysis.

Table S4: GO term analysis for each of the clusters (Related to Fig. 4).

Table includes the GO Biological processes, molecular function, cellular component and KEGG pathway analysis for each of the clusters.

Table S5: Protein groups that are significantly regulated between young and aged OPCs (Related to Fig.6).

Table showing the log_2 change, and q value of the 659 proteins differentially expressed between young and aged OPCs. Data also include the GO terms enriched with the 659 proteins differentially expressed between young and aged OPCs as well as the GO terms enriched for those proteins that are upregulated or downregulated.

Table S6: All proteins detected in the Multiplex 1 of the ageing OPC proteome (Related to Fig.1).

Table showing all the proteins detected in the first multiplex and their identification characteristics detected by Proteome Discoverer.

Table S7: All proteins detected in the Multiplex 2 of the ageing OPC proteome (Related to Fig.1).

Table showing all the proteins detected in the second multiplex and their identification characteristics detected by Proteome Discoverer.
